# The Dorsal Raphe Regulates the Duration of Attack through the Medial Orbitofrontal Cortex and Medial Amygdala

**DOI:** 10.1523/ENEURO.0331-20.2020

**Published:** 2020-10-22

**Authors:** Jacob Nordman, Zheng Li

**Affiliations:** 1 Section on Synapse Development Plasticity, National Institute of Mental Health, National Institutes of Health, Bethesda, MD 20892; 2 National Institute of General Medical Sciences, National Institutes of Health, Bethesda, MD 20892

**Keywords:** aggression, dorsal raphe, medial amygdala, neurocircuit, optogenetics, orbitofrontal cortex

## Abstract

The dorsal raphe (DR) is an evolutionarily conserved brain structure that is involved in aggressive behavior. It projects onto numerous cortical and limbic areas underlying attack behavior. The specific neurocircuit through which the DR regulates aggression, however, is largely unclear. In this study we show that DR neurons expressing CaMKIIα are activated by attack behavior in mice. These neurons project to the medial aspect of the orbitofrontal cortex (OFC; MeOC) and the medial amygdala (MeA), two key regions within the neural circuit known to control aggressive behavior. Using an *in vivo* optogenetic approach, we show that attack bouts are shortened by inhibiting CaMKIIα^+^ neurons in the DR and their axons at the MeOC and prolonged by stimulating the DR-MeOC axons during an attack. By contrast, stimulating the axons of CaMKIIα^+^ DR neurons at the MeA shortens attack. Notably, neither the DR-MeOC or DR-MeA pathway initiates attack when stimulated. These results indicate that the DR-MeOC and DR-MeA pathways regulate the duration of attack behavior in opposite directions, revealing a circuit mechanism for the control of attack by the DR.

## Significance Statement

The dorsal raphe (DR) is a major node in the brain circuit regulating multiple attack behaviors. The underlying neurocircuitry through which the DR acts on aggression, however, remains elusive. Here, we show that the DR regulates the duration of attack through the medial orbitofrontal cortex (OFC; MeOC) and the medial amygdala (MeA), areas known to play a key role in aggression. While neither pathway is sufficient to initiate an attack, silencing the DR-MeOC pathway or activating the DR-MeA pathway shortens an attack, and stimulation of the DR-MeOC circuit prolongs an already occurring attack. These findings identify two DR-mediated neural circuits that regulate attack behavior.

## Introduction

The dorsal raphe (DR) nucleus is one of the raphe nuclei located on the midline of the brainstem. It is a phylogenetically conserved structure and plays a role in various types of aggressive behaviors, such as maternal and territorial aggression in rodents (Walletschek and Raab, 1982; Takahashi and Miczek, 2014; Holschbach et al., 2018; Muroi and Ishii, 2019). The role of the DR in aggression is complex and context dependent. For example, infusion of glutamate in the DR increases the frequency of attack bites against a conspecific, but with no effect on threatening behavior (Takahashi et al., 2015). Conversely, infusion of glutamate receptor agonists increases bite latency and decreases bite frequency in maternal aggression, with no effect on chasing behavior (Muroi and Ishii, 2019). Knock-down of tyrosine receptor kinase receptors in DR neurons decreases latency to attack (Adachi et al., 2017). Prepartum lesion of the DR decreases the frequency of attack in maternal aggression, while postpartum lesion of the DR decreases the duration of an individual attack bout (Holschbach et al., 2018).

The DR contains a heterogenous population of neurons that release one or a combination of the neurotransmitters serotonin (5-hydroxytryptamine or 5-HT), dopamine, glutamate and GABA (Liu et al., 2014; Ren et al., 2018; Huang et al., 2019). Glutamatergic neurons of the DR innervate dopamine neurons of the ventral tegmental area (VTA) to reinforce instrumental responding and establish conditioned place preference (Qi et al., 2014). GABAergic interneurons of the DR mediate the acquisition of avoidance after social defeat, as demonstrated by optogenetic silencing of the GABAergic input to local 5-HT neurons (Challis et al., 2013). Dopaminergic neurons of the DR are required for rebound sociability after social isolation (Matthews et al., 2016). The serotonergic neurons of the DR are involved in aggressive behavior. In mice with reduced 5-HT release at the DR, defensive but not offensive aggression increases as determined by counter-attack bites (Chen et al., 1994). In maternal aggression, activation of 5-HT1A somatodendritic autoreceptors in the DR promotes lateral attacks, but with no effect on threatening behavior (da Veiga et al., 2011). Studies linking the DR to aggression are largely confined to the 5-HT neurons (Olivier, 2004; Nelson, 2006; Nelson and Trainor, 2007). The role of other DR cell types in aggression is unclear.

The prefrontal cortex and amygdala are densely innervated by the DR (Wilson and Molliver, 1991; Clarke et al., 2007; Ren et al., 2018). The orbitofrontal cortex (OFC) and medial amygdala (MeA) are subregions within these areas that receive DR inputs (Cádiz-Moretti et al., 2016; Murphy and Deutch, 2018; Ren et al., 2018) and regulate intermale aggression (Blair, 2004; Siever, 2008; Rosell et al., 2010; Hong et al., 2014; Rosell and Siever, 2015; Unger et al., 2015; Buades-Rotger et al., 2017; Haller, 2018). DR neurons projecting to the OFC and the MeA may control attack behavior in different ways. In support of this hypothesis, 5-HT signaling, the majority of which derives from neurons in the raphe, at the OFC and MeA appears to have different effects on attack behavior. In subsets of individuals with personality disorder that exhibit impulsive aggression 5-HT2A expression is increased at the OFC, as determined by positron emission tomography of a 5-HT2AR radioligand, while infusion of agonists of 5-HT1B autoreceptors, which inhibits 5-HT release, at the OFC suppresses attack behavior in mice (De Almeida et al., 2006; Rosell et al., 2010). Conversely, stimulation of 5-HT2A receptors in the MeA suppresses shock-induced attack behavior and muricide, while inhibition of 5-HT2 receptors enhances it (Rodgers, 1977; Puciłowski et al., 1985). These findings suggest that DR input promotes attack at the OFC but suppresses attack at the MeA. A direct manipulation of the DR projections to the OFC and MeA is necessary to tease apart their specific roles in aggressive behavior.

Using an *in vivo* optogenetic approach, we show that CaMKIIα^+^ neurons in the DR are activated by attack and that these neurons modulate the duration of attack behavior toward an intruder through two projection areas. Specifically, the DR-medial OFC (MeOC) pathway prolongs an already occurring attack, while the DR-MeA pathway shortens it. These findings reveal two DR-mediated neurocircuits that have divergent functions in aggressive behavior.

## Materials and Methods

### Animals and reagents

All animal protocols were approved by the Animal Care and Use Committee (ACUC). Five-week-old C57BL/6 male mice were purchased from Charles River and housed under a 12-h light (9 P.M. to 9 A.M.)/12-h dark (9 A.M. to 9 P.M.) cycle with *ad libitum* access to water and food. All mice were individually housed for three weeks before testing to increase aggression (Malick, 1979; Valzelli, 1985). Smaller, submissive, male mice group housed with littermates were used as intruders to promote aggressive behavior in resident mice (Koolhaas et al., 2013). Intruder mice did not attack during aggression tests in this study. Reagents are listed in Table 1.

**Table 1.** Key resources table

Resource type	Specific reagent or resource	Source or reference	Identifiers	Dilution or concentration
Organism/strain	C57BL/6J	Charles River	Strain code: 556	
Antibody	CaMKIIa (Cba-2) mouse monoclonal antibody	Abcam	Catalog #137300	1:250 dilution
Antibody	c-Fos rabbit polyclonal antibody	Abcam	Catalog #ab190289	1:2000 dilution
Antibody	GFP polyclonal antibody	MBL	Catalog #598	1:1000 dilution
Antibody	Anti-TpH2 antibody	Abcam	Catalog #ab184505	1:500 dilution
Antibody	Alexa Fluor 488 goat anti-mouse IgG	ThermoFisher	Catalog #A-10680	1:200 dilution
Antibody	Alexa Fluor 555 goat anti-mouse IgG	ThermoFisher	Catalog #A-21422	1:200 dilution
Antibody	Alexa Fluor 488 goat anti-rabbit IgG	ThermoFisher	Catalog #A-11034	1:200 dilution
Antibody	Alexa Fluor 555 goat anti-rabbit IgG	ThermoFisher	Catalog #A-21428	1:200 dilution
Bacterial or viral strain	AAV2/9.CaMKIIa (1.3 kb). hChR2 (E123A)-mCherry.WPRE. hGH	Addgene	Catalog #35505-AAV9	≥1 × 10¹³ vg/ml, 500 nl injected
Bacterial or viral strain	AAV2/9.CaMKIIa (1.3 kb). hChR2 (E123A)-eYFP.WPRE. hGH	Addgene	Catalog #35506-AAV9	≥1 × 10¹³ vg/ml, 500 nl injected
Bacterial or viral strain	AAV2/9.CaMKIIa (1.3 kb).ArchT 3.0-eYFP.WPRE. hGH	Addgene	Catalog #99039-AAV9	≥1 × 10¹³ vg/ml, 500 nl injected
Bacterial or viral strain	pRRlsin.eGFP	In house		
Commercial assay or kit	Metabond	Parkell	Catalog #S380	
Commercial assay or kit	Dental cement	DuraLay	Catalog #602-7395	
Commercial assay or kit	Vectashield HardSet Antifade Mounting Medium with DAPI	Vector Laboratories	Catalog #H-1500	
Software; algorithm	SigmaPlot	IBM	https://www.ibm.com	

### Surgery

Six- to seven-week-old C57BL/6 male mice were anaesthetized with isoflurane (3% for induction and 1% for maintenance) and then placed onto a stereotaxic frame (David Kopf Instruments). Craniotomy was made and 500 nl virus (AAV2/9-CaMKIIα (1.3 kb variant)-ChR2 (E123A)-mCherry, AAV2/9-CaMKIIα (1.3 kb variant)-ChR2 (E123A)-EYFP, AAV2/9-CaMKIIα (1.3 kb variant)-ArchT-EYFP or the lentiviral vector pRRlsin.CMV:eGFP as control virus) was injected into the center of the DR (beginning at skull surface, bregma coordinates: –4.3 mm AP, 1.10 mm ML, –2.85 mm DV, 20° ML angle) using a 5-μl gas-tight Hamilton syringe (33-gauge, beveled needle) at a rate of 75 nl/min. The ChR2 (E123A)-EYFP used in this study is the ultrafast opsin variant ChETA(A) (Gunaydin et al., 2010; Mattis et al., 2011). After injection, the needle was left in place for an additional 5 min and then slowly withdrawn. After viral injection, ferrule-terminated optical fibers (100 μm in diameter, ThorLabs) were placed 100 μm above the viral injection site at the DR or bilaterally above the MeOC (beginning at skull surface, bregma coordinates: +2.4 mm AP, +/−1.7 mm ML, –1.7 mm DV) or the MeA (beginning at skull surface, bregma coordinates: −1.5 mm AP, +/−2.1 mm ML, −5 mm DV). Optical fibers were secured to the skull using Metabond (Parkell), stainless steel screws (PlasticsOne) and dental cement (DuraLay). After surgery, mice recovered on a heated pad until ambulatory and then were returned to their home cage for six weeks before optical stimulation.

### Resident intruder (RI) test

Before the RI test, mice were individually housed for three weeks. All behavioral experiments took place during the dark cycle of the day, as this is the main activity phase of the mouse (Koolhaas et al., 2013). On the day of testing, mice were transferred in their home cage to a behavioral test room and allowed to acclimate for at least 1 h. Younger, group-housed target conspecific males were placed into the home cage of the resident mouse and the two were allowed to freely interact for 10 min. All animals were allowed to habituate to the patch cord for 20 min before introduction of the conspecific. Baseline aggression was tested at 1–4 d before the RI test. Animal behavior was captured with a video camera. If excessive tissue damage occurred, the test was prematurely terminated and not analyzed. Excessively aggressive mice, as determined by total attack time >40% during the RI test, were eliminated from further analysis (Hong et al., 2014; Nordman et al., 2020a). Videos of behavioral tests were reviewed and hand scored by a researcher blind to the experimental conditions using a bin size of 0.5 s. Aggressive behaviors were identified as chasing, boxing, pinning, biting, and wrestling (Blanchard and Blanchard, 1977; Lin et al., 2011; Koolhaas et al., 2013; Hong et al., 2014; Golden et al., 2016).

### *In vivo* optogenetic stimulation

Optogenetic stimulation was performed via an optical fiber (ferrule fiber, ThorLabs) connected through a zirconia split sleeve and patch cord to a 473 nm laser (Coherent) or a 561 nm laser (CrystaLaser) under the control of an Optogenetics TTL Pulse Generator (Doric Lenses). Mice expressing ChR2 were stimulated for 5 ms using 1- to 3-mW 473 nm light pulsed at 10 Hz for 10 s. Mice expressing ArchT were delivered a 1- to 3-mW continuous 10-s 561 nm light pulse. Laser was manually turned on and the frequency and duration of light pulses were controlled by Doric Studio software (Doric).

### Immunohistochemistry

Mice were transcardially perfused with 4% paraformaldehyde in PBS solution. Brains were removed and postfixed at 4°C overnight, then cryoprotected overnight in 15% sucrose (in PBS) followed by 30% sucrose in PBS. Brains were cut into 30-μm-thick sections using a cryostat (Leica CM3050-S), then either mounted onto silanized slides (KD Medical) or stored in PBS as floating sections for immunohistochemistry or confirming the location of viral injection and implantation. For immunohistochemistry, free floating brain sections were heated to 80°C for 30 min in citrate buffer for antigen retrieval (Jiao et al., 1999) and then blocked with 10% goat serum and 1% bovine serum albumin in PBS with 0.03% Triton X-100 (PBS-T) for 3 h at room temperature. Sections were then stained for primary antibodies overnight at 4°C, followed by incubation with secondary antibodies for 1 h at room temperature. Sections were mounted to slides with Vectashield HardSet Antifade Mounting Medium containing DAPI.

### Image acquisition and analysis

Brain slices were imaged with a multi-slide fluorescent microscope (Zeiss Axio Scan) with a 10× (NA 0.45) objective to locate the areas with fluorescence signals, and then a laser scanning confocal microscope (Zeiss LSM510 and LSM780) with a 40× (NA 1.3 oil immersion) objective for high-magnification imaging in the region of interest. Z-stack confocal images were collapsed and analyzed with ImageJ by a researcher blind to the experimental conditions. c-Fos positive cells were identified using the “Analyze Particles” function of ImageJ and validated as cells by their overlap with DAPI. Cells co-labeled for DAPI and CaMKIIα and/or c-Fos were manually counted by a researcher blind to the experimental conditions.

### Statistical analysis

All data were presented as individual data points and mean ± SEM SigmaPlot software was used for statistical analysis. Data were tested for normality and equal variance. Student’s *t* test (for data that satisfied normal distribution and equal variance) and Mann–Whitney *U* test (for data that did not satisfy normal distribution and equal variance) were used to compare two groups and one-way ANOVA was used to test for differences among three groups. Tukey’s test was used for *post hoc* multiple comparisons to identify groups that were significantly different; *p* < 0.05 was considered significant and all tests were two tailed. All statistical data can be found in Table 2.

**Table 2:** Statistical table

Data	Method	Factor	*n*	*T*, *U*, or *F* stat	*p* value	*Post hoc* correction
Fig. 1*C*	Mann–Whitney	Resident vs control	6, 7	*U* = 2	0.008	
Fig. 1*D*	Mann–Whitney	Resident vs control	6, 7	*U* = 5	0.027	
Fig. 1*E*	Student’s *t* test	Resident vs control	6, 7	*T*_(11)_ = 0.164	0.872	
Fig. 1*F*	Student’s *t* test	Resident vs control	6, 7	*T*_(11)_ = 0.062	0.952	
Fig. 1*H*	One-way ANOVA	# of cells at bregma −4.84	5, 5	*F*_(2,14)_ = 13.339	<0.001	Tukey’s
	One-way ANOVA	# of cells at bregma −4.60	5, 5	*F*_(2,14)_ = 6.357	0.013	Tukey’s
	One-way ANOVA	# of cells at bregma −4.34	5, 5	*F*_(2,14)_ = 168.490	<0.001	Tukey’s
Fig. 1*I*	One-way ANOVA	% of DAPI at bregma −4.84	5, 5	*F*_(2,14)_ = 13.859	<0.001	Tukey’s
	One-way ANOVA	% of cells at bregma −4.60	5, 5	*F*_(2,14)_ = 4.493	0.035	Tukey’s
	One-way ANOVA	% of cells at bregma −4.34	5, 5	*F*_(2,14)_ = 20.455	<0.001	Tukey’s
Fig. 2*C*	Mann–Whitney	ChR2 vs GFP	6, 6	*U* = 0	0.005	
Fig. 2*D*	Student’s *t* test	ChR2 vs GFP	6, 6	*T*_(10)_ = 0.203	0.843	
Fig. 2*G*	One-way ANOVA	TpH2 vs ChR2	5, 5	*F*_(2,17)_ = 13.714	<0.001	Tukey’s
	One-way ANOVA	TpH2 vs ArchT	5, 5	*F*_(2,17)_ = 20.868	<0.001	Tukey’s
Fig. 2*K*	Student’s *t* test	Opsin vs GFP	5, 4	*T*_(7)_ = 0.793	0.454	
Fig. 2*O*	Student’s *t* test	Before light onset (10 s)	5, 4	*T*_(7)_ = −0.607	0.563	
	Student’s *t* test	During light (10 s)	5, 4	*T*_(7)_ = 0.882	0.407	
Fig. 2*S*	Student’s *t* test	Before light onset (10 s)	5, 3	*T*_(6)_ = 0.136	0.896	
	Student’s *t* test	During light (10 s)	5, 3	*T*_(6)_ = −10.784	<0.001	
Fig. 3*L*	Student’s *t* test	ChR2 vs GFP at bregma 2.68	6, 6	*T*_(10)_ = 0.325	0.752	
	Student’s *t* test	ChR2 vs GFP at bregma 2.68	6, 6	*T*_(10)_ = 0.890	0.346	
	Student’s *t* test	ChR2 vs GFP at bregma 2.34	6, 6	*T*_(10)_ = 0.396	0.701	
Fig. 3*M*	Student’s *t* test	ChR2 vs GFP at bregma 2.34	6, 6	*T*_(10)_ = 8.033	<0.001	
	Student’s *t* test	ChR2 vs GFP at bregma 2.10	6, 6	*T*_(10)_ = 3.850	0.003	
	Student’s *t* test	ChR2 vs GFP at bregma 2.10	6, 6	*T*_(10)_ = 7.965	<0.001	
Fig. 4*L*	Student’s *t* test	ChR2 vs GFP	6, 6	*T*_(10)_ = 0.474	0.646	
Fig. 4*M*	Student’s *t* test	ChR2 vs GFP	6, 6	*T*_(10)_ = 0.558	0.589	
Fig. 4*N*	Student’s *t* test	ChR2 vs GFP	6, 6	*T*_(10)_ = 0.258	0.801	
Fig. 4*O*	Student’s *t* test	ChR2 vs GFP	6, 6	*T*_(10)_ = 1.100	0.297	
Fig. 4*P*	Student’s *t* test	ChR2 vs GFP	6, 6	*T*_(10)_ = 1.203	0.257	
Fig. 4*Q*	Student’s *t* test	ChR2 vs GFP	6, 6	*T*_(10)_ = 4.693	<0.001	
Fig. 4*R*	Student’s *t* test	ChR2 vs GFP	6, 6	*T*_(10)_ = 2.745	0.021	
Fig. 4*S*	Student’s *t* test	ChR2 vs GFP	6, 6	*T*_(10)_ = 3.104	0.011	
Fig. 4*T*	Student’s *t* test	ChR2 vs GFP	6, 6	*T*_(10)_ = 1.597	0.141	
Fig. 4*U*	Student’s *t* test	ChR2 vs GFP	6, 6	*T*_(10)_ = 2.525	0.030	
Fig. 5*D*	Student’s *t* test	Opsin vs GFP	4, 3	*T*_(5)_ = 1.101	0.321	
Fig. 5*H*	Student’s *t* test	Before light onset (10 s)	4, 3	*T*_(5)_ = 0.845	0.437	
	Mann–Whitney	During light (10 s)	4, 3	*U* = 28	0.952	
Fig. 5*L*	Student’s *t* test	Before light onset (10 s)	4, 3	*T*_(5)_ = 0.632	0.555	
	Student’s *t* test	During light (10 s)	4, 3	*T*_(5)_ = −6.606	<0.001	
Fig. 6*D*	Student’s *t* test	Opsin vs GFP	5, 4	*T*_(7)_ = 0.137	0.895	
Fig. 6*H*	Student’s *t* test	Before light onset (10 s)	4, 3	*T*_(5)_ = 0.845	0.437	
	Mann–Whitney	During light (10 s)	4, 3	*T*_(5)_ = 0	1.000	
Fig. 6*L*	Student’s *t* test	Before light onset (10 s)	6, 4	*T*_(8)_ = −1.217	0.278	
	Student’s *t* test	During light (10 s)	6, 4	*T*_(8)_ = 0.743	0.491	
Fig. 7*D*	Student’s *t* test	Opsin vs GFP	4, 3	*T*_(5)_ = 1.130	0.310	
Fig. 7*H*	Student’s *t* test	Before light onset (10 s)	4, 3	*T*_(5)_ = 1.425	0.214	
	Student’s *t* test	During light (10 s)	4, 3	*T*_(5)_ = 8.871	<0.001	
Fig. 7*L*	Student’s *t* test	Opsin vs GFP	6, 4	*T*_(8)_ = 1.010	0.342	
Fig. 7*P*	Student’s *t* test	Before light onset (10 s)	6, 4	*T*_(8)_ = 0.229	0.826	
	Student’s *t* test	During light (10 s)	6, 4	*T*_(8)_ = −12.538	<0.001	

## Results

**Figure 1.**
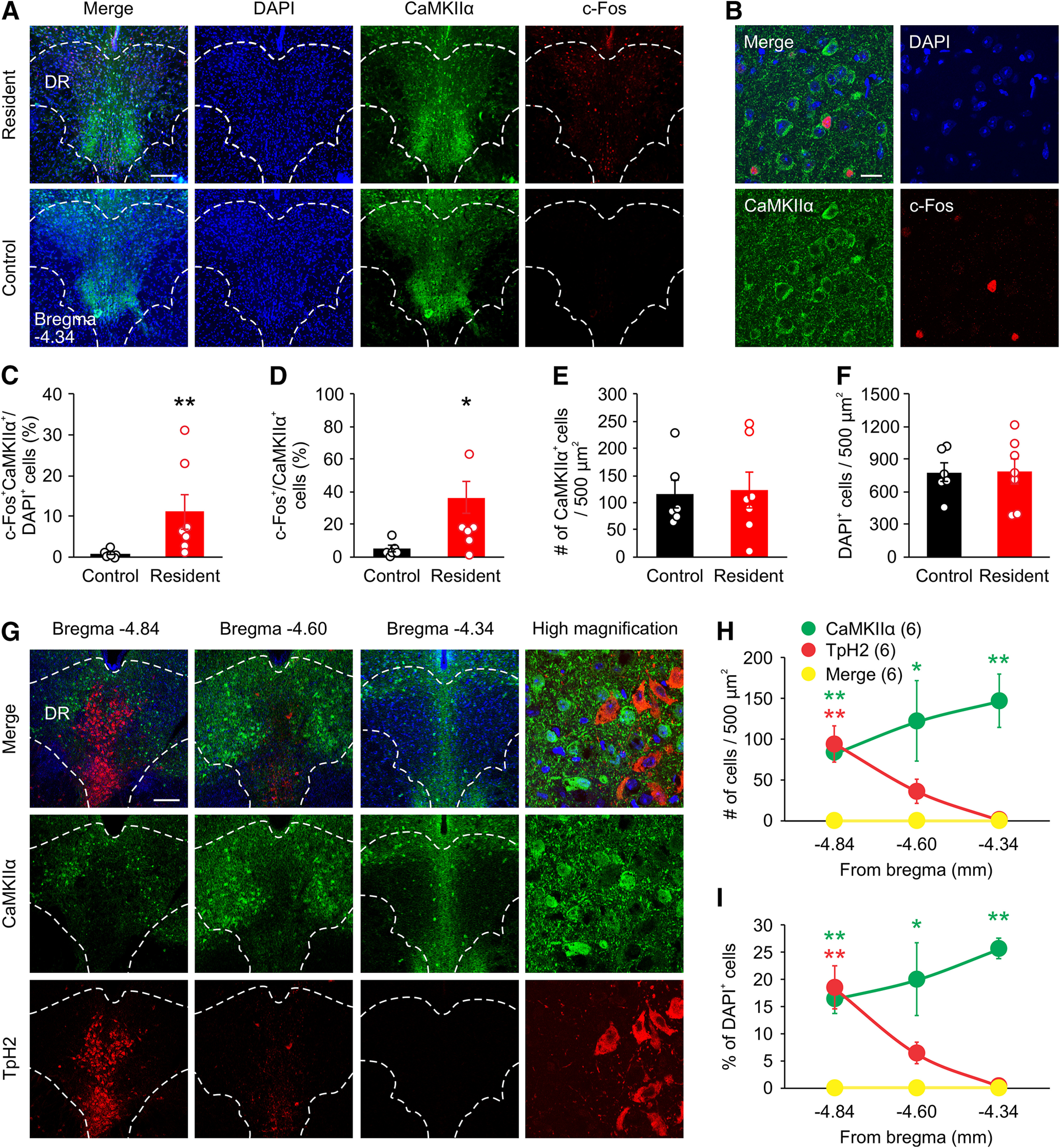
CaMKIIα^+^ neurons of the DR are activated by attack. ***A–F***, Mice (10 weeks of age) were examined for aggression using the RI assay. Mice were perfused 60 min after the assay for immunostaining. ***A***, Representative images of brain sections stained for c-Fos and CaMKIIα in the DR from resident or control animals 1 h after the RI test. ***B***, Representative high-magnification images of CaMKIIα^+^ cells in the DR that were positive for c-Fos. ***C***, ***D***, Percentage of c-Fos^+^ cells that colocalize with CaMKIIα^+^ and DAPI^+^ cells in resident and control mice for ***A***. ***E***, ***F***, Quantification of CaMKIIα^+^ (***E***) and DAPI^+^ (***F***) cells in the DR in resident and control mice. ***G***, Representative images of brain sections stained for CaMKIIα and TpH2 throughout the DR (bregma −4.34 to −4.84 mm). High-magnification image of the DR is taken from a brain slice at bregma position −4.84, as this is where the majority of TpH2^+^ cells can be found. ***H***, Quantification of total number of CaMKIIα^+^ and TpH2^+^ cells in the DR. Asterisks indicate statistical significance of CaMKIIα (green) or TpH2 (red) from merged set at the specified coordinate. One slice was quantified per area per animal. Animal number is indicated in parentheses. ***I***, Percentage of DAPI^+^ cells in the DR that co-label for CaMKIIα and TpH2. Asterisks indicate statistical significance of CaMKIIα (green) or TpH2 (red) from merged set at the specified coordinate. Only cells within the DR were counted. Scale bars: 200 μm (***A***, ***G***) and 25 μm (***B***). Data are presented as mean ± SEM; *p < 0.05, **p < 0.01. Statistics can be found in Table 2.

Excitatory neurons have been found in the DR (Commons, 2009; Qi et al., 2014; Zhou et al., 2015; Ren et al., 2018; Huang et al., 2019) and implicated in aggression (Chen et al., 1994). To better assess their function in aggressive behavior, we exposed C57BL/6 mice (male, 10 weeks of age) to the RI test and stained brain sections of the resident mice with antibodies against c-fos, which labels activated neurons, and CaMKIIα, a protein primarily expressed by excitatory neurons but not GABAergic neurons in many brain regions (Benson et al., 1992; Jones et al., 1994; Liu and Jones, 1996). The number of cells within the DR that were doubly positive for CaMKIIα and c-Fos significantly increased after the RI test, suggesting that CaMKIIα^+^ cells are activated by attack behavior (Fig. 1*A–D*). The total number of CaMKIIα^+^ and DAPI^+^ cells within the DR was comparable in resident and control mice (Fig. 1*E*,*F*). Because many DR cells co-release 5-HT and glutamate (Liu et al., 2014; Ren et al., 2018; Huang et al., 2019), we co-stained the DR sections for CaMKIIα and the 5-HT cell marker tryptophan hydroxylase type 2 (TpH2). No CaMKIIα^+^ cells co-localized with TpH2 throughout the DR, indicating that CaMKIIα^+^ neurons in the DR are not serotonergic (Fig. 1*G-I*).

**Figure 2.**
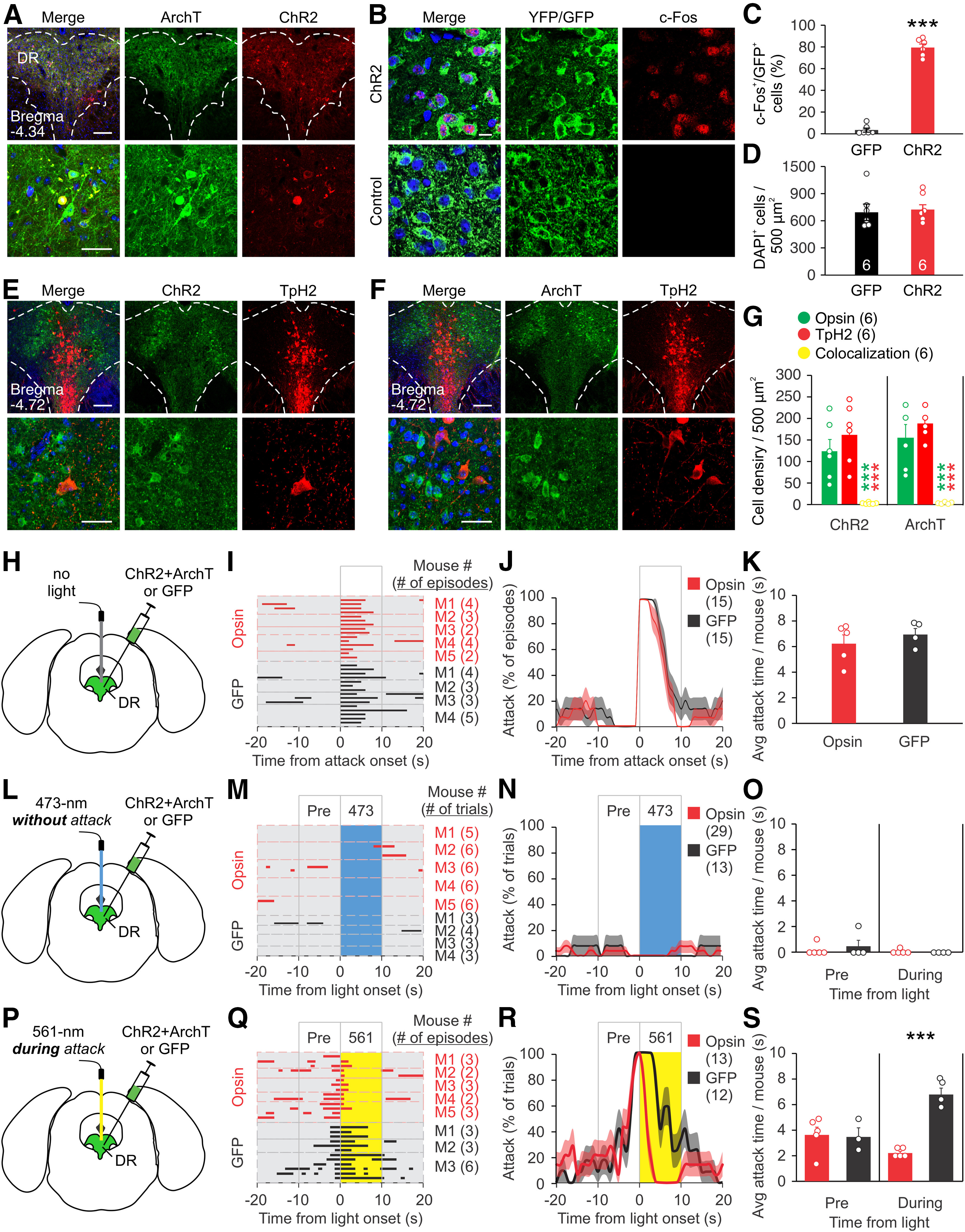
Inhibition of CaMKIIα^+^ DR neurons shortens attack. ***A***, Representative high-magnification and low-magnification images of ChR2-mCherry and ArchT-EYFP expression in the DR three weeks after viral injection. ***B***, Representative images of brain sections stained for c-Fos in the DR of mice injected with ChR2-EYFP or GFP and photostimulated with 473 nm light (pulsed at 10 Hz for 10 s). ***C***, ***D***, Percentage of YFP^+^ or GFP^+^ cells that colocalize with c-Fos (***C***) and quantification of total DAPI^+^ cells (***D***) in the DR after photostimulation for ***B***. Only cells within the DR were counted. ***E***, ***F***, Representative images of brain sections expressing ChR2 (***E***) or ArchT (***F***) under the CaMKIIα promoter co-stained for TpH2 in the DR. ***G***, Quantification of ChR2 or ArchT (opsin) expressing cells that were stained positive for TpH2. Only cells within the DR were counted. One slice was quantified per area per animal. Animal number is indicated in parentheses. ***H***, ***L***, ***P***, Schematic drawing of viral injection (AAV expressing ChR2 and ArchT or GFP control virus), placement of optic fiber, and stimulation procedure. Opsin expressing mice in ***H–K*** and opsin stimulated mice in L-S were injected with ChR2 virus and ArchT virus. The same mice were stimulated with 473 or 561 nm light on separate days. ***I***, Raster plots of attack events during each interaction episode (rows in the raster plot, defined as the period from 20 s before to 20 s after the onset of a spontaneous attack) for each mouse. All attack events during the testing period are shown. ***J***, % of episodes (rows) in which mice attacked at each time point in I; red and dark lines indicate the mean, pink and gray areas indicate SEM. ***K***, Quantification of average attack time per mouse during the 10 s after the onset of a spontaneous attack, as represented in the boxed area in ***J***. ***M***, Raster plots of trials (rows) of mice photostimulated with 473 nm light at the DR when the mouse was not attacking. All trials are aligned to the onset of light. ***N***, % of trials in which mice attacked at each time point in ***M***; red and dark lines indicate the mean, pink and gray areas indicate SEM. ***O***, Quantification of attack time per mouse before and during light stimulation for ***M***. ***Q***, Raster plots of trials (rows) of mice photostimulated with 561 nm light at the DR during an attack. All trials are aligned to onset of light. ***R***, % of trials in which mice attacked at each time point in ***Q***; red and dark lines indicate the mean, pink and gray areas indicate SEM. ***S***, Quantification of attack time per mouse before and during light stimulation for ***Q***. Scale bars: 200 μm (low-magnification image; top; ***A***, ***E***, ***F***); 50 μm (high-magnification image; bottom; ***A***, ***E***, ***F***); 10 μm (***B***). Data are presented as mean ± SEM; ***p < 0.001. Statistics can be found in Table 2.

To determine the function of CaMKIIα^+^ DR neurons in aggressive behavior, we opted for an optogenetic approach to alter their activities. Eight-week-old mice were injected with AAV expressing ChR2-mCherry (for neural activation) and ArchT-EYFP (for neural inhibition) under the CaMKIIα promoter into the DR for use in the RI test three weeks later. Overlapping expression of ChR2 and ArchT was detected in the DR (Fig. 2*A*). Photostimulation of the DR using 473 nm light (5-ms light pulsed at 10-Hz for 10 s) activated the DR neurons transduced with ChR2-EYFP as determined by c-Fos staining (Fig. 2*B–D*), indicating that photostimulation is effective. Previous studies have found that the CaMKIIα promoter in AAV vectors drives protein expression primarily in excitatory cells but in a few inhibitory neurons as well (Scheyltjens et al., 2015; Watakabe et al., 2015). We detected few cells transduced with ChR2 or ArchT under the CaMKIIα promoter that were TpH2^+^ (Fig. 2*E–G*), consistent with our finding that the CaMKIIα^+^ cells of the DR are not serotonergic (Fig. 1*G–I*).

We first assessed baseline aggression in mice expressing ChR2 and ArchT or GFP control virus without light stimulation. Attack duration was comparable in ChR2/AchT and GFP mice, suggesting that expression of ChR2 and ArchT has no effect on baseline aggression (Fig. 2*H–K*). To test the effect of light stimulation on aggression, light pulses were delivered to the DR; 473 nm light stimulation applied when the animal was not attacking did not increase attack behavior for the duration of the light pulse (Fig. 2*L–O*). However, 561 nm light stimulation (10-s constant light) delivered after attack had begun significantly reduced attack duration during the light pulse (Fig. 2*P–S*). Attack behavior during the 10-s prestimulation period did not differ between opsin and control mice (Fig. 2*M–O*,*Q–S*). These results indicate that inhibition of CaMKIIα^+^ DR neurons suppress ongoing attack.

### CaMKIIα^+^ DR neurons project to the MeOC and MeA to regulate aggression

**Figure 3.**
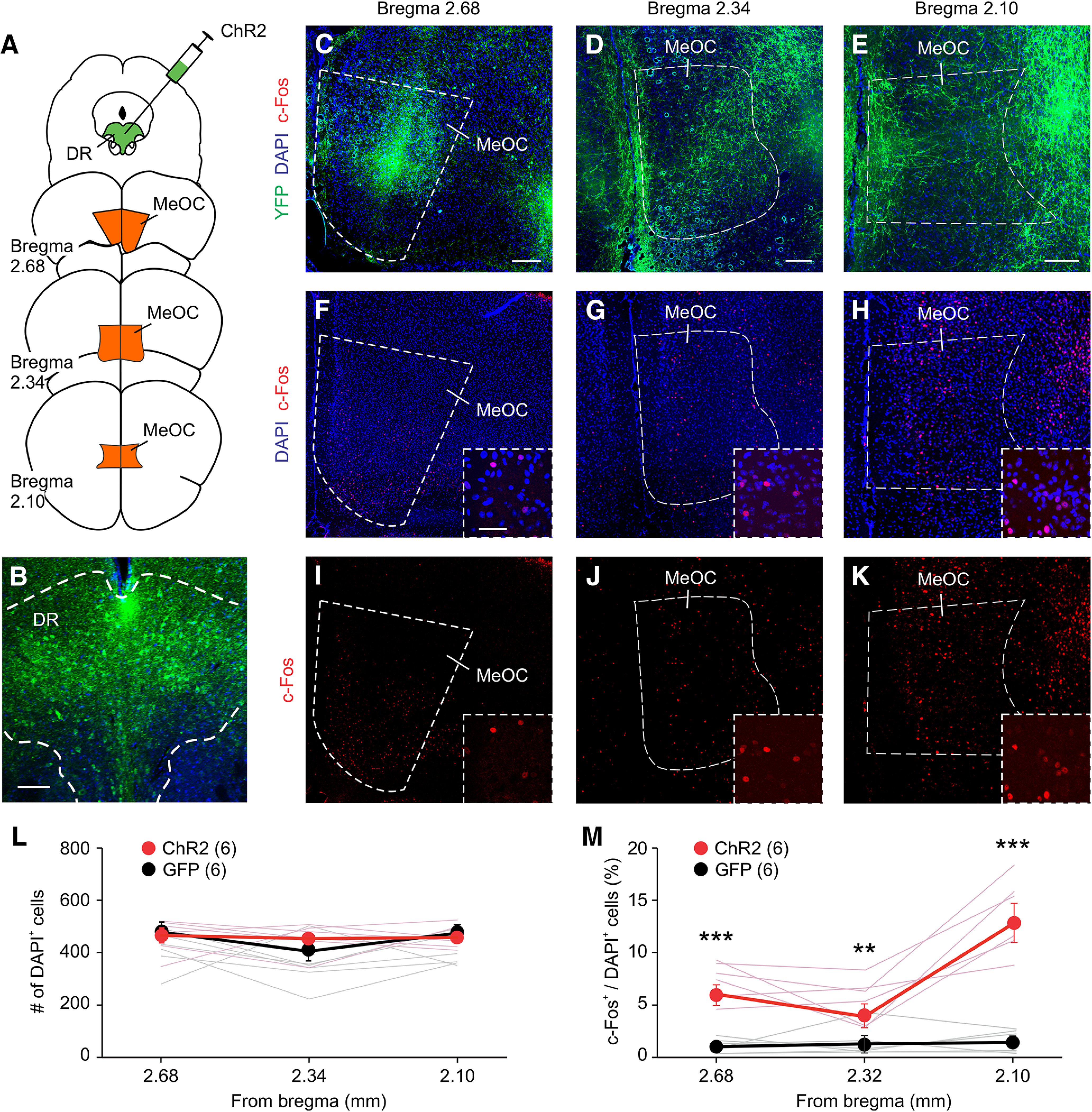
DR neurons project to and activate the MeOC. ***A–E***, Schematic drawing of ChR2-EYFP viral injection and representative images of ChR2 expression in DR neurons (***B***) and DR projections at the MeOC (***C–E***) three weeks later. ***F–K***, c-Fos labeling of mice photostimulated at the DR-MeOC. ***L***, ***M***, Quantification of total number of cells (***L***) and percentage (***M***) of c-Fos^+^ cells in the MeOC for ***D–K***. Only cells within the MeOC were counted. One slice was quantified per area per animal. Animal number is indicated in parentheses. Scale bars: 200 μm (***A***, ***B***; low-magnification images in ***C***, ***D***) and 50 μm (high-magnification images in ***C***, ***D***). Data are presented as mean ± SEM; **p < 0.01, ***p < 0.001. Statistics can be found in Table 2.

**Figure 4.**
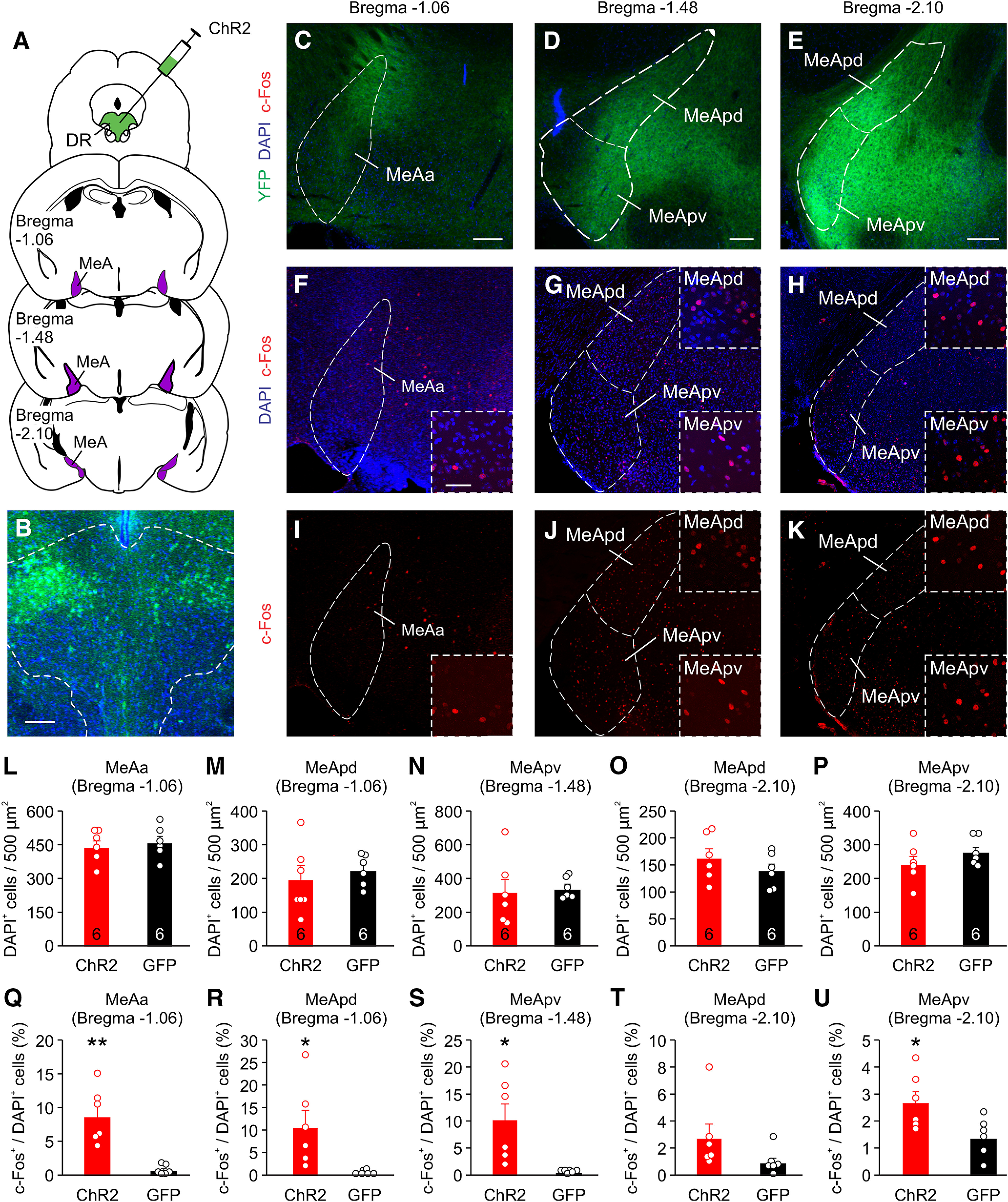
DR neurons project to and activate the MeA. ***A–E***, Schematic drawing of ChR2-EYPF injections into the DR and representative images of ChR2 expression in DR neurons (***B***) and axons at the MeA (***C–E***) three weeks later. ***F–K***, c-Fos labeling of mice photostimulated at the DR-MeA. ***L–U***, Quantification of total number of cells (***L–M***) and percentage (***Q–U***) of c-Fos^+^ cells in the MeA (MeAa, anterior MeA; MeApd, posteriordorsal MeA; MeApv, posteriorventral MeA) for ***D–K***. Only cells within the MeA were counted. One slice was quantified per area per animal. Animal number is indicated in bars of bar graphs. Scale bars: 200 μm (***A***); 200 μm (low-magnification images of ***B–D***, low-magnification images of ***F–K***); 50 μm (high-magnification images of ***F–K***). Data are presented as mean ± SEM; *p < 0.05, **p < 0.01. Statistics can be found in Table 2.

To investigate the circuit mechanism by which CaMKIIα^+^ DR neurons regulate attack behavior, we stimulated the DR axons in the OFC and MeA, two DR projection areas (Cádiz-Moretti et al., 2016; Murphy and Deutch, 2018; Ren et al., 2018) involved in aggressive behavior (Blair, 2004; Siever, 2008; Rosell et al., 2010; Hong et al., 2014; Rosell and Siever, 2015; Unger et al., 2015; Buades-Rotger et al., 2017; Haller, 2018). Mice were injected with AAV expressing ChR2-EYFP into the DR (Figs. 3*A*,*B*, 4*A*,*B*) and then examined for YFP expression in the OFC and MeA. YFP^+^ DR axons were found in the MeOC and throughout the MeA (Figs. 3*C–E*, 4*C–E*). Photostimulation of the DR using 473 nm light (5-ms pulsed at 10 Hz for 10 s) activated cells within both regions as determined by c-Fos staining (Figs. 3*F–M*, 4*F–U*). The MeA can be divided into three main subdivisions: the anterior MeA (MeAa), the posteriordorsal MeA (MeApd), and the posteriorventral MeA (MeApv), all of which are involved in aggressive behavior (Kollack-Walker and Newman, 1995; Lin et al., 2011; Hong et al., 2014; Miller et al., 2019; Nordman et al., 2020a). Analysis of c-Fos expression in the MeA revealed that all three regions are activated by photostimulation of the DR (Fig. 4*L–U*).

**Figure 5.**
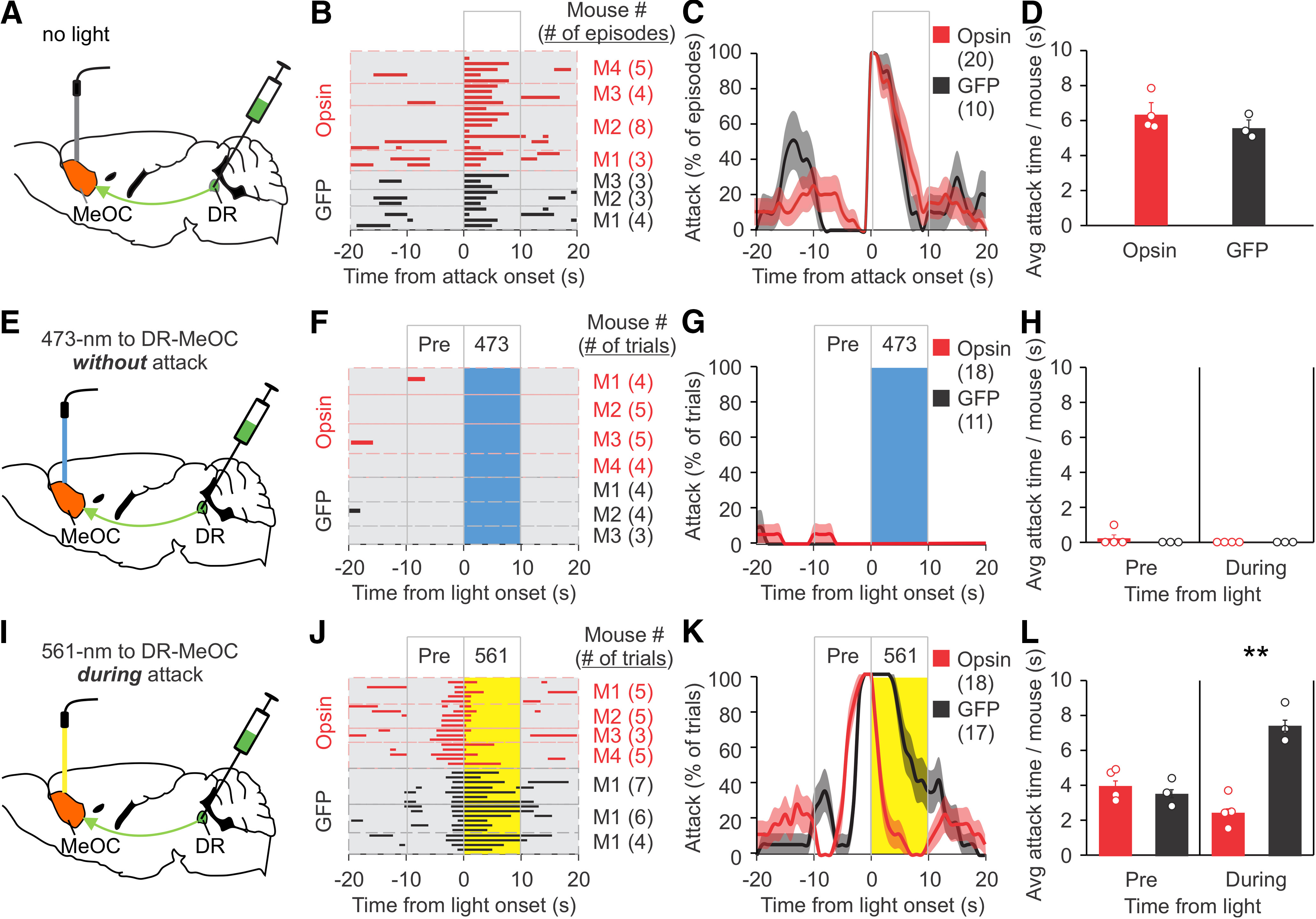
Inhibition of CaMKIIα^+^ DR input to the MeOC shortens attack. ***A***, ***E***, ***I***, Schematic drawing of viral injection (AAV expressing ChR2 and ArchT or GFP control virus), placement of optic fiber, and stimulation procedure. Opsin expressing mice in ***A–D*** and opsin stimulated mice in ***E–L*** were injected with ChR2 virus and ArchT virus. The same mice were stimulated with 473 or 561 nm light on separate days. ***B***, Raster plots of attack events during each interaction episode (rows in the raster plot, defined as the period from 20 s before to 20 s after the onset of a spontaneous attack) for each mouse. All attack events during the testing period are shown. ***C***, % of episodes (rows) in which mice attacked at each time point in ***B***; red and dark lines indicate the mean, pink and gray areas indicate SEM. ***D***, Quantification of average attack time per mouse during the 10 s after the onset of a spontaneous attack, as represented in the boxed area in ***C***. ***F***, Raster plots of trials (rows) of mice photostimulated with 473 nm light at the DR-MeOC when the mouse was not attacking. ***G***, % of trials in which mice attacked at each time point in ***F***; red and dark lines indicate the mean, pink and gray areas indicate SEM. ***H***, Quantification of attack time per mouse before and during light stimulation for ***F***. ***J***, Raster plots of trials (rows) of mice photostimulated with 561 nm light at the DR-MeOC during an attack. ***K***, % of trials in which mice attacked at each time point in ***J***; red and dark lines indicate the mean, pink and gray areas indicate SEM. ***L***, Quantification of attack time per mouse before and during light stimulation for ***J***. Data are presented as mean ± SEM; **p < 0.01. Statistics can be found in Table 2.

**Figure 6.**
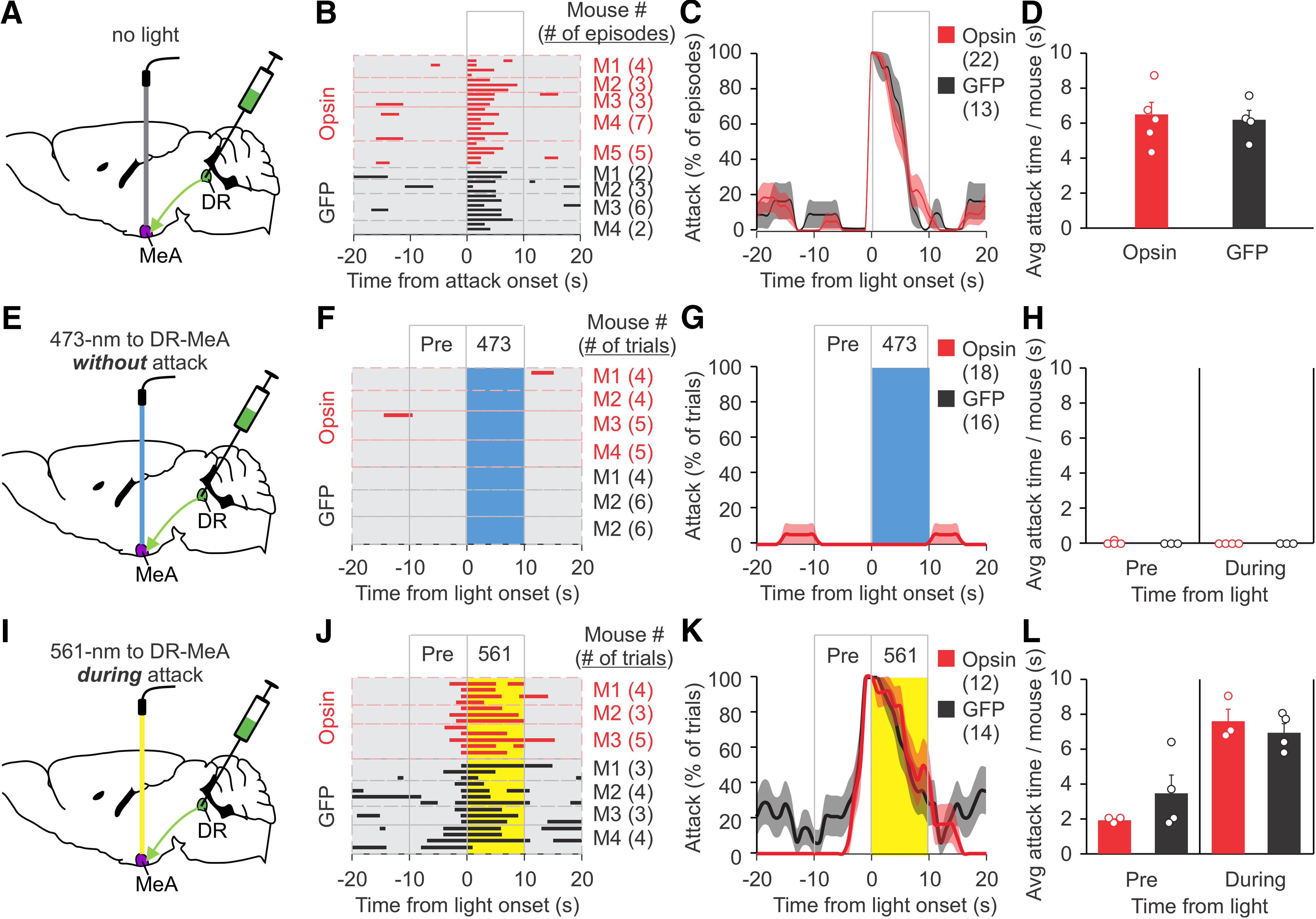
Effects on attack of CaMKIIα^+^ DR input to the MeA. ***A***, ***E***, ***I***, Schematic drawing of viral injection (AAV expressing ChR2 and ArchT or GFP control virus), placement of optic fiber, and stimulation procedure. Opsin expressing mice in ***A–D*** and opsin stimulated mice in ***E–L*** were injected with ChR2 virus and ArchT virus. The same mice were stimulated with 473 or 561 nm light on separate days. ***B***, Raster plots of attack events during each interaction episode (rows in the raster plot, defined as the period from 20 s before to 20 s after the onset of a spontaneous attack) for each mouse. All attack events during the testing period are shown. ***C***, % of episodes (rows) in which mice attacked at each time point in ***B***; red and dark lines indicate the mean, pink and gray areas indicate SEM. ***D***, Quantification of average attack time per mouse for the 10 s after the onset of a spontaneous attack, as represented in the boxed area in ***C***. ***F***, Raster plots of trials (rows) of mice photostimulated with 473 nm light at the DR-MeA when the mouse was not attacking. ***G***, % of trials in which mice attacked at each time point in ***F***; red and dark lines indicate the mean, pink and gray areas indicate SEM. ***H***, Quantification of attack time per mouse before and during light stimulation for ***F***. ***J***, Raster plots of trials (rows) of mice photostimulated with 561 nm light at the DR-MeA during an attack. ***K***, % of trials in which mice attacked at each time point in J; red and dark lines indicate the mean, pink and gray areas indicate SEM. ***L***, Quantification of attack time per mouse before and during light stimulation for ***J***. Data are presented as mean ± SEM. Statistics can be found in Table 2.

**Figure 7.**
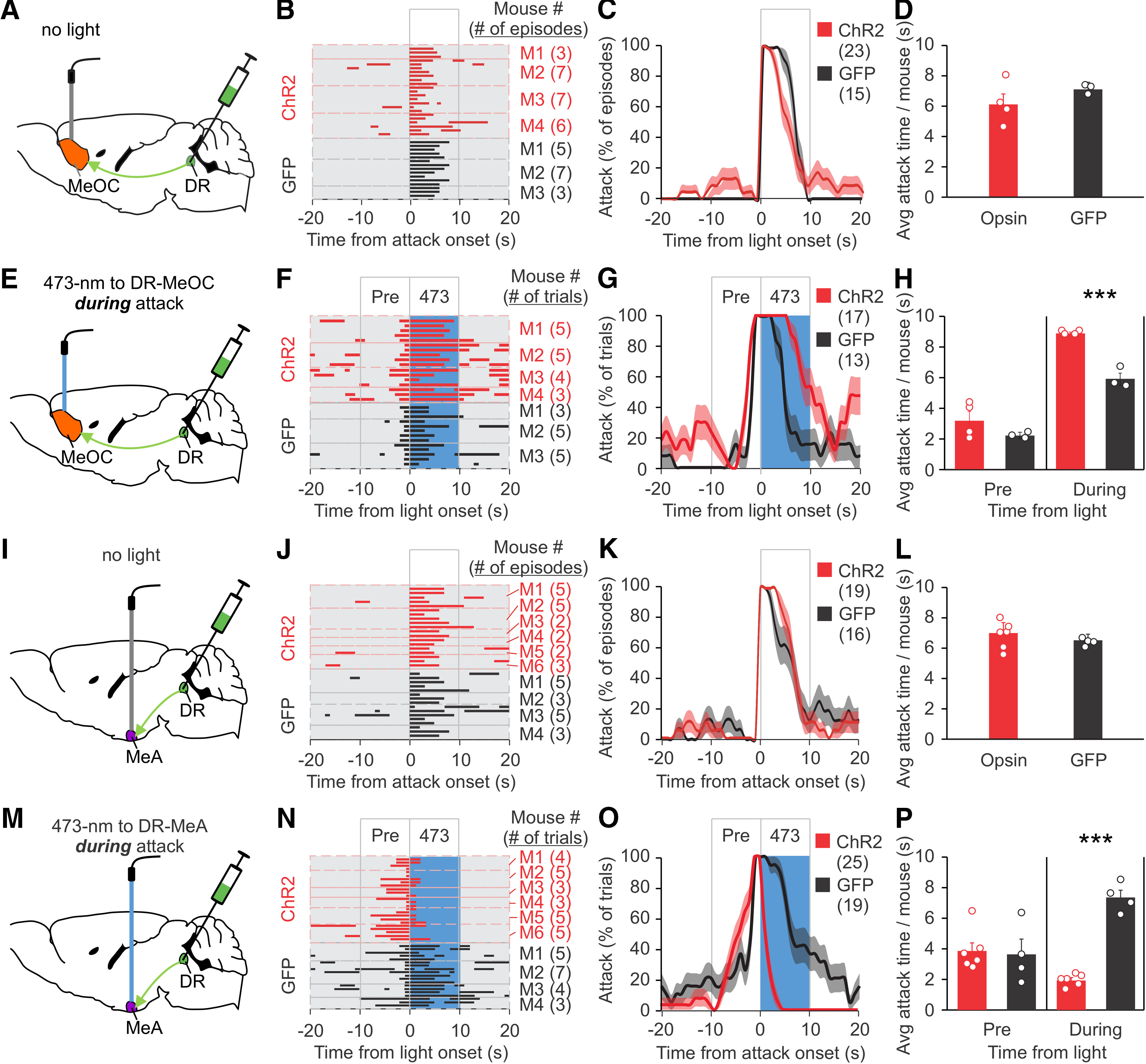
Activation of the DR-MeOC prolongs attack and activation of the DR-MeA shortens attack. Mice were injected with ChR2 virus or GFP virus into the DR and optical fibers were placed into the MeOC (***A***, ***E***) or the MeA (***I***, ***M***). RI tests were performed eight weeks later. Separate groups of mice were used for ***A–H*** and ***I–P***. ***B***, Raster plots of attack events during each interaction episode (rows in the raster plot, defined as the period from 20 s before to 20 s after the onset of a spontaneous attack) for each mouse. All attack events during the testing period are shown. ***C***, % of episodes (rows) in which mice attacked at each time point in ***B***; red and dark lines indicate the mean, pink and gray areas indicate SEM. ***D***, Quantification of average attack time per mouse for the 10 s after the onset of a spontaneous attack, as represented in the boxed area in ***C***. ***F***, Raster plots of trials (rows) of mice photostimulated with 473 nm light at the DR-MeOC during an attack. ***G***, % of trials in which mice attacked at each time point in F; red and dark lines indicate the mean, pink and gray areas indicate SEM. ***H***, Quantification of attack time per mouse before and during light stimulation for ***F***. ***J***, Raster plots of attack events during each interaction episode (rows in the raster plot, defined as the period from 20 s before to 20 s after the onset of a spontaneous attack) for each mouse. All attack events during the testing period are shown. ***K***, % of episodes (rows) in which mice attacked at each time point in ***J***; red and dark lines indicate the mean, pink and gray areas indicate SEM. ***L***, Quantification of average attack time per mouse for the 10 s after the onset of a spontaneous attack, as represented in the boxed area in ***J***. ***N***, Raster plots of trials (rows) of mice photostimulated with 473 nm light at the DR-MeA during an attack. ***O***, % of trials in which mice attacked at each time point in ***N***; red and dark lines indicate the mean, pink and gray areas indicate SEM. ***P***, Quantification of attack time per mouse before and during light stimulation for ***N***. Data are presented as mean ± SEM; ***p < 0.001. Statistics can be found in Table 2.

DR neurons were injected with ChR2-mCherry and ArchT-EYFP virus and then implanted with optical fibers into the MeOC or MeA (Figs. 5*A*,*E*,*I*, 6*A*,*E*,*I*). The RI test was performed eight weeks later. Baseline attacks in opsin and GFP mice were comparable (Figs. 5*B–D*, 6*B–D*). During the RI test, mice were stimulated with 473 nm light when not attacking or 561 nm light after attack had begun. As with the DR, 473 nm light stimulation at the MeOC or MeA did not increase attack behavior (Figs. 5*E–H*, 6*E–H*). While 561 nm light had no effect on attack behavior when applied to the MeA, it shortened attack time when applied to the MeOC (Figs. 5*I–L*, 6*I–L*). The ChR2/ArchT and GFP control groups had comparable attack behavior before light stimulation (Figs. 5*F–H*,*J–L*, 6*F–H*,*J–L*). These results suggest that the input from DR CaMKIIα^+^ neurons to the MeOC is required for attack to continue. Furthermore, stimulating the DR axons at the MeOC during an attack with 473 nm light pulses significantly increased attack duration, again with no differences in baseline attacks or in attack behavior during the pre-light stimulation period (Fig. 7*A–H*). These results indicate that the DR-MeOC pathway can prolong an already occurring attack.

Stimulation of the MeA has been shown to suppress aggression (Rodgers, 1977; Puciłowski et al., 1985; Hong et al., 2014). To assess whether stimulation of the input from DR CaMKIIa1 neurons to the MeA may inhibit ongoing attack, we stimulated the MeA of mice injected with AAV ChR2 into the DR at the onset of an attack (Fig. 7*M*); 473 nm photostimulation of the MeA significantly shortened the duration of an attack during illumination. Baseline and prestimulation attack behaviors were comparable in ChR2 and GFP control mice (Fig. 7*I–P*). These results suggest that the DR-MeA pathway facilitates the termination of attack.

Taken together, these findings indicate that the projections from the CaMKIIα^+^ DR neurons to the MeOC and MeA have opposite effects on attack behavior, with the DR-MeOC inputs sustaining attack while the DR-MeA inputs shorten attack.

## Discussion

The DR modulates attack behavior during aggressive encounters in rodents (Chen et al., 1994; Takahashi et al., 2010, 2015; da Veiga et al., 2011; Adachi et al., 2017; Balázsfi et al., 2018; Muroi and Ishii, 2019). The DR contains a heterogenous population of neurons that project to various brain regions to control social behavior and emotion (Challis et al., 2013; Qi et al., 2014; Matthews et al., 2016; Ren et al., 2018; Huang et al., 2019). However, the role of specific DR projections in aggressive behavior is incompletely understood. Here, we show that the DR-MeOC and DR-MeA pathways control attack duration in opposite directions.

The MeOC and MeA are subregions densely innervated areas by the DR (Cádiz-Moretti et al., 2016; Murphy and Deutch, 2018; Ren et al., 2018) and are key nodes in the processing of social interaction including aggression (Blair, 2004; Siever, 2008; Rosell et al., 2010; Hong et al., 2014; Rosell and Siever, 2015; Unger et al., 2015; Buades-Rotger et al., 2017; Haller, 2018). Activity within the OFC is negatively correlated with aggression and chronic inactivation or lesioning in this area heightens aggression in mice and humans (Anderson et al., 1999; Blair, 2004; Siever, 2008; Rosell et al., 2010; Beyer et al., 2015; Rosell and Siever, 2015; Kuniishi et al., 2016). The role of the MeA in aggression is better characterized. Within the MeA, activation of GABAergic neurons in the MeApd promote attack, while activation of glutamatergic neurons suppress it (Hong et al., 2014; Padilla et al., 2016). Stimulation of dopamine D1 receptor (D1R)-expressing neurons within the MeApv projecting to the bed nucleus of the stria terminalis increases aggression, while stimulation of those projecting to the ventromedial hypothalamus decreases aggression (Miller et al., 2019). Potentiation of synapses between the MeApv and the ventromedial hypothalamus and bed nucleus of the stria terminalis underlies aggression priming and heightened aggression induced by traumatic stress (Nordman et al., 2020a,b). Notably, dysfunction within the OFC and MeA is associated with excessive and impulsive aggression in mice and humans (Grafman et al., 1996; Blair, 2004; New et al., 2004; Shalom et al., 2004; Coccaro et al., 2007; Mpakopoulou et al., 2008; Buades-Rotger et al., 2017; Herpertz et al., 2017). In this study, we chose to stimulate the axons of the CaMKIIα^+^ DR neurons at the MeOC and MeA to discriminate the effects of different pathways on attack behavior. We show that optogenetic silencing of CaMKIIα^+^ neurons in the DR and their projections to the MeOC reduces the duration of an attack while optogenetic activation of the DR-MeOC prolongs it. Conversely, activation of the DR-MeA projections reduces attack duration. These results raise the possibility that distinct groups of CaMKIIα^+^ DR neurons project to the MeOC and MeA. It is interesting that stimulating the DR-MeOC pathway mimics the effect of stimulating the DR on aggression, suggesting that the DR-MeOC pathway predominates over the DR-MeA pathway. It is noted that the MeA receives direct inputs from the OFC (Siever, 2008; Márquez et al., 2013; Cádiz-Moretti et al., 2016), leaving open the possibility that the DR-MeA pathway is suppressed by OFC input when the DR-MeOC pathway is activated.

The CaMKIIα promoter has been extensively used to drive gene expression in excitatory neurons, though it is also active in a small number of inhibitory neurons in the cortex (Scheyltjens et al., 2015; Watakabe et al., 2015). In addition, many glutamatergic cells of the DR co-release serotonin (Liu et al., 2014; Ren et al., 2018; Huang et al., 2019). Thus, one limitation of our study is that our AAV with the CaMKIIα promoter may transduce non-excitatory neurons in the DR. It is noted that in a previous study stimulation of DR neurons that were transduced with AAV expressing ChR2 under the pan neuronal promoter synapsin lead to a decrease in aggression and an increase in 5-HT and GABA release at the PFC (Balázsfi et al., 2018). These synapsin promoter targeted neurons likely overlap with the CaMKIIα^+^ promoter targeted neurons in this study. This raises an intriguing possibility that the CaMKIIα^+^ DR neurons may release neurotransmitters other than glutamate to regulate aggressive behavior. Our finding that the CaMKIIα^+^ DR neurons do not overlap with the serotonergic cell marker TpH2 would suggest that the CaMKIIα^+^ cells are non-serotonergic (Figs. 1*G–I*, 2*E–G*). The release of other neurotransmitters, however, cannot be ruled out.

The three main MeA subregions, MeAa, MeApd, and MeApv, are all involved in aggressive behavior (Kollack-Walker and Newman, 1995; Lin et al., 2011; Hong et al., 2014; Miller et al., 2019; Nordman et al., 2020b). While we observed that photostimulation of the DR can activate all three subregions, since the MeApd and MeApv have different effects on aggression (Kollack-Walker and Newman, 1995; Lin et al., 2011; Hong et al., 2014; Miller et al., 2019; Nordman et al., 2020a), it is worth considering that the DR may be shortening an attack through a specific subregion of the MeA. The photostimulation protocol used in this study does not allow for discrimination of these subregions because of their anatomic clustering.

Aggression is an adaptive behavior with the intention of preserving resources or protecting oneself from harm. Excessive aggression, however, is energetically unfavorable (Maynard Smith and Price, 1973; Haller, 1995; Miczek et al., 2013). In mice, prolonged attack is an indicator of excessive aggression (Miczek et al., 2013). The neural circuits that underlie prolonged aggression are poorly defined. Our study demonstrates that while neither the DR-MeOC or DR-MeA pathway can initiate an attack, both pathways regulate the duration of an already occurring attack. These findings suggest an intriguing possibility that dysfunction within these DR pathways may play a role in excessive aggression.

## Synthesis

Reviewing Editor: Carmen Sandi, Swiss Federal Institute of Technology Decisions are customarily a result of the Reviewing Editor and the peer reviewers coming together and discussing their recommendations until a consensus is reached. When revisions are invited, a fact-based synthesis statement explaining their decision and outlining what is needed to prepare a revision will be listed below. The following reviewer(s) agreed to reveal their identity: Mate Toth.

This is an intriguing study addressing specific questions in the field of aggressive behavior. Specifically, the manuscript describes a novel divergent non-serotonergic pathway from the dorsal raphe nucleus to the medial orbitofrontal cortex and to the medial amygdala (MeA) which differentially modulates. The authors investigated behavioral relevance of a novel divergent non-serotonergic pathway from the dorsal raphe (DR) nucleus to the medial orbitofrontal cortex (MeOC) and to the medial amygdala (MeA). While many previous studies focused on serotonergic modulation emanating from the DR, in this manuscript the authors studied the role of axonal projections from the CaMKII-positive cells, which most likely mediate glutamatergic excitatory transmission in the target areas. Interestingly, using bidirectional optogenetic manipulations in-vivo of the cell bodies in the DR and of the axonal projections in the MeOC and the MeA during resident-intruder aggression test, the authors found a target-specific differential modulation of the aggression bout duration. Namely, activation or inhibition of the DR to MeOC projections increased or, respectively, decreased the duration of aggression, while stimulation of the DR to MeA projections decreased the duration of aggression (no effect of inhibition). Notably, this effect was observed only on ongoing aggression, and neither of the pathways triggered aggression upon stimulation. Therefore, the study shows that DR CaMKII neurons control attack behavior via the orbitofrontal cortex, without initiating the actual attack. In contrast, these neurons exert an opposite effect in the medial amygdala. These data suggest a complex coordinated control over aggressive behavior by the raphe. These findings are novel, intriguing and timely because the mechanisms of how aggressive behavior is exactly regulated are very complex and we are still far from detailed understanding of the network mechanisms. The reviewers and editor consider that this work can be a nice contribution to this growing field.

The design and results seem to be quite clear and robust. However, there are several concerns that need to be addressed regarding the data presentation, control experiments, data analysis and statistics. Although the number of issues raised below seems long and complex, we believe that the authors have the necessary data and material to address them for a revision of the manuscript. The specific issues are:

Main points:

1. Histological analysis showing that CaMKII cells and TpH2 cells do not overlap is incomplete. Fig. 1: When comparing the images in Fig. 1A and Fig. 1G, it becomes apparent that these slices were taken from different AP positions. One can appreciate it from the different, almost complementary pattern of CaMKII staining between 1A and 1G: in the former, CaMKII cells are grouped more medially and ventrally, while in the latter - scattered more laterally and dorsally. This notion is strengthened by strikingly different quantification results in Fig. 1E vs Fig. 1H: around 120 cells / 500 um2 (correct? - see minor point #18) in 1E versus about 40 cells per 1000 um2 in 1H (i.e. 6 times lower density of CaMKII cells than in Figs. 1G-I). If so, then it is important to know how TpH2 positive cells are distributed at the AP level corresponding to Fig. 1A: if they are distributed similarly as in Fig. 1G, would there be more co-localization between CaMKII and TpH2? To address these questions, please consider adding more overview images to the Fig. 1A and 1G to exemplify both AP levels in both panels, and please indicate the approximate AP level in mm according to an atlas. Also, it is important to know how many slices per animal were used for the cell counting - did the quantified slices cover the whole extend of DR along the rostro-caudal axis? Please report the average number of slices analyzed per animal and the AP ranges they covered, in the results or in the figure legend.

2. CaMKII promoter used in the viral vectors does not guarantee specificity of expression in general, and it is not known how it behaves in the DR. Fig. 2: A general problem with using promoters in the viral vectors as compared to the Cre mouse lines is that the promoters are often not specific leading to off-target expression in undesired cell types (e.g. see Scheyltjens et al., 2015 J Comp Neurol; Watakabe et al. 2015 Neurosci Res for nonspecific expression of CaMKII promoter viruses in inhibitory cells of the cortex). The degree of non-specificity depends on many factors such as AAV serotype, viral titer, promoter variant and the brain region (therefore these parameters should be reported in the Methods, see minor point #7). For this particular study, claiming the role for non-serotonergic DR neurons in behavior, it is thus quite important to report that the serotonergic neurons were at least largely spared from the opsin expression under conditions used for in-vivo experiments. As a part of Fig. 2 the authors should provide immunohistological results of TpH2 staining in brain slices after injection with AAV expressing both opsins and report the degree of colocalization, which is crucial for correct interpretation of their results.

3. Concurrently with the (critically) small number of animals used in behavior experiments (n=3 to max 5 per group), there are issues of possible experimental bias during measurements, and the statistical analysis was not designed appropriately (pooling of trials, comparison between groups of small size with potentially high inter-subject variability). Figs. 2, 4, 5, behavioral analysis:

A) A borderline-small number of mice (only 3 or 4, in some cases 5) were used in behavioral experiments. It is also not clear if the same mice were used between ChR2 and ArchT exeriments e.g. in Fig. 4, because the authors used co-injections of ChR2 and ArchT constructs at least in some experiments (Fig. 2). If animals were shared between different experiments in the Fig. 2E-H and 2I-L, and Fig. 4A-D and E-H, Fig. 4I-L, M-P, it should be clearly reported in the figure legends.

B) For the statistical analysis, several aggression trials (i.e. technical replicates) from different mice were pooled together for a two-sample statistical test. The total number of trials was ranging from 11 to 29, while correspondence to individual animals was not reported, and the trials were plotted as individual data points in the bar graphs in Figs. 2L, 4H,P, 5D,H. This pooling results in artificially small error bars and high statistical significance. Individual mice may have strongly varying aggressiveness: if an animal is too aggressive, it will have many long-lasting aggressive bouts, and vice versa. If pooling is unbalanced, this can introduce bias and thus is not acceptable. Instead, the mean duration of aggression in each individual mouse should be treated as one data point for statistical comparison. Alternatively, one can use a so-called “nested t-test” available in GraphPad Prism, which is very similar to that.

C) Why do individual trial measurements in the bar graphs (Figs. 2L, 4H,P 5D,H) acquire only integer values, i.e. 1, 2, 3 etc. seconds? This limitation was not explained in the methods, and conventional video recording should normally feature much higher time resolution for behavioral scoring.

D) From the raster plots shown in Figs. 2J, 4F, 5F it seems that light for GFP group was in most cases turned on very soon or immediately after the start of aggression bout. However, in case of ArchT/ChR2 groups, up to a few seconds were allowed to pass after the onset of aggression bouts before the light went on. This also can be appreciated from the traces in Fig. 4G, 5G (not so evident in 2H). Effectively, this bias leads to reduction of measured aggression duration during light, which is the main reported effect in these experiments. Why did this kind of bias take place? One possibility is that the experimenter pressing the button to start the optostimulation, was not blind to experimental condition and waited longer after aggression onset, more often for ArchT/ChR2 group than for GFP group. Could the authors comment on that? The good news is that, despite this observation, the reported effect seems to be quite robust at the level of pooled trials. Nevertheless, I think it is necessary to additionally address this issue in order to counteract possible concerns, which could be done e.g. as proposed below in (E).

E) As mentioned above, the number of experimental animals used in this study is quite small, with possible big differences between individuals, thus comparison between the ArchT/ChR2 and GFP groups is risky. To complement the existing analysis, it would be very useful to compare behavior before and during the light within the same animals to normalize for individual differences. Since direct comparison of total duration of aggression cannot be done due to the experimental design (because the light was turned on specifically during aggression bouts), one possibility would be to compare the distributions and/or statistics of distributions of the aggression bout durations for “spontaneous” attacks in between the light pulses, with those affected by the light pulses. This should be done in each animal to obtain, e.g. a ratio between the mean duration of spontaneous and light-affected aggression bouts.

Minor points:

1. Please specify primary antibodies.

2. Please clarify experimental settings in terms of light presentation. What were exactly ‘before light’ and ‘during light’ conditions and how were they defined? E.g. all attacks occuring during no light periods vs all attacks during light exposure? Then related question: how were light pulses scheduled, i.e. predefined or manually initiated when an attack occured (for all or some, which were the ‘no light’ trials then, every other one e.g.)?

3. For the interested reader, it would be useful to have a detailed desciption of DR-OFC and DR-MeA projection (AP distribution with additional representative photographs).

4. Line 32: the reference of Adachi et al., (2017) is wrongly cited. At least in its published form, that paper showed a decrease in latency to attack upon deletion (genetic knockdown) of TrkB receptor, and not upon its activation. Effect on the number of attacks was however not directly reported. Please bring the text in correspondence with the cited publication.

5. Line 34: This unformatted citation is wrong and most probably should cite another paper by Takahashi et al., J Neuroci (2015).

6. Line 61: Citation of Ren et al. 2018 is imprecise since that study investigated projections from the DR to OFC and CeA (not MeA). By the way, none of the OFC or CeA projectors seemed to significantly collaterize to the MeA as shown in Fig. 2 therein. Thus, to avoid misleading citation, this sentence has to be reformulated, or another reference relevant for the MeA should be added.

7. A statement and the block of citations, lines 68-71: A) The study by Shih et al (1999) used ketanserin and MDL100907, both being 5-HT2AR antogonists, in MAO A mutant mice that have elevated 5-HT concentration. Thus, this citation rather contradicts the main point of the sentence. B) The study by Sakaue et al (2002) showed the effect of 5HT2A activation (DOI) or inhibition (ritanserin) acting in exactly opposite directions (increasing or decreasing aggression, respectively) than stated. C) In general, the studies that used systemic applications of agonists/antagonists, including Shih et al 1999, Sakaue et al 2002, should not be cited in the context of any specific brain region. D) Citation of a book by Nelson (2006) is not acceptable to support such a specific statement. E) To my knowledge, crickets used as a model animal by Rillich and Stevenson 2018 do not have a medial amygdala, therefore please either remove or cite it in a different context.

8. Lines 87-88: “Smaller, submissive intruder mice” - were these intruders pre-screened to determine their hierarchy position? Please specify.

9. Citations block, lines 89-91: The cited studies dealt with either male or female animals at a time and thus naturally focused at sex-specific types of aggression, which are basically different behaviors. Resident-intruder test is classically male-specific behavioral model and needs no further explanations. The authors may rather mention the influence of social isolation, sexual experience, hierarchical status on aggression in males with respective references, and thus explain the use of virgin, singly housed, pre-screened, etc. animals.

10. Lines 95-96: Please provide the AAV serotype, final titer and the provider (with catalog number if available) of each viral vector, and also completely spell out the GFP control vector. Was the full-length or short CaMKII promoter used? Was ChR2 a H134R mutant?

11. Line 97: Please specify which “0” level was used for AP and DV coordinates: i.e. bregma, skull surface?

12. Line 122: Please state if the mice were previously habituated to the procedure of optic patch cord attachment and to the head tethering with the optic fiber, or not.

13. Line 126: For 10 Hz stimulation, please specify the pulse duration.

14. Lines 138-139: Please list all the primary and the secondary antibodies used (with the species, suppliers, catalog numbers and the dilutions used).

15. Lines 163-164: For better readability it would be better to provide only relevant citations for separate parts of the statement. For example, in this citation block only few references dealt with aggression, while the others studied different cell types in the DR without making a link to aggression. Altogether the sentence in its current form is misleading if citations are expected to provide relevant argumentation.

16. Line 169: Is the study by Wallen-Mackenzie et al 2009 really relevant here to support the statement?

17. Line 180: In the Methods, ArchT construct is referred to as ArchT-EYFP and not as ArchT-GFP as here. Please homogenize.

18. Lines 190-191: To prove this sufficiency statement, a stimulation experiment in DR during attack would be necessary which is then expected to prolong the attack bouts (as in Fig. 5). In case the authors performed this experiment, it should be reported in the text.

19. Citations in the lines 215-216: The two first studies used serotonin to stimulate the MeA, and its effect on MeA neurons (excitatory or inhibitory) is not known, it depends on the receptor type. In contrast, Hong et al., 2014, showed either strong activation or suppression of aggression, depending on stimulation regime (high/low power) and the cell type (VGAT/VGlu). The use of these citations to support this particular statement is thus misleading.

20. Discussion: The authors should cite the previous report by Balázsfi et al. 2018 that used partially overlapping optogenetic settings: DR of C57Bl6 mice was infected with AAV2.5 to express ChR2 under hsyn promoter. Most likely, this approach also led to stimulation of CaMKII positive neurons in the DR, and thus the authors should discuss their results in respect with the results of that previous report.

21. Fig. 1E, axis label: should it be the cell density (per 500 um2)?

22. Fig 2J and line 505 in its figure legend: Should be ArchT, not ChR2

23. Fig. 2B: description in the Methods, and the results in Fig. 2A say that ChR2 should have an mCherry tag. Why is then in Fig. 2B (top row) ChR2 co-colocalizes with GFP? Was here another construct (ChR2-GFP) used, as in Fig. 3? If so, please make the description clearer in the text and please list the ChR2-GFP construct in the Methods.

24. Fig. 3: This figure represents a quite important experiment, and the important details which should be available to the authors seem to be under-reported. As in Fig. 1, the authors should report the AP coordinates for the quantified slices and the number of slices analyzed per animal. In the case of MeA, it would be quite easy to separately report the cell density (or % of DAPI cells) for the ventral and the dorsal MeA. Optionally, if the data is already available or easy to obtain, it would be very interesting to know which fraction of cFos-positive cells in the ventral and dorsal MeA is GABAergic and which is glutamatergic. This knowledge is crucial for interpretation of the results of this study in the light of previous reports on the role of different MeA cell populations in aggression control (e.g. Hong et al. 2014; Miller et al. 2019 Nature Neurosci - not cited here).

## References

[B1] Adachi M, Autry AE, Mahgoub M, Suzuki K, Monteggia LM (2017) TrkB signaling in dorsal raphe nucleus is essential for antidepressant efficacy and normal aggression behavior. Neuropsychopharmacology 42:886–894. 10.1038/npp.2016.201 27634357 PMC5312065

[B2] Anderson SW, Bechara A, Damasio H, Tranel D, Damasio AR (1999) Impairment of social and moral behavior related to early damage in human prefrontal cortex. Nat Neurosci 2:1032–1037. 10.1038/14833 10526345

[B3] Balázsfi D, Zelena D, Demeter K, Miskolczi C, Varga ZK, Nagyváradi Á, Nyíri G, Cserép C, Baranyi M, Sperlágh B, Haller J (2018) Differential roles of the two raphe nuclei in amiable social behavior and aggression - an optogenetic study. Front Behav Neurosci 12:163. 10.3389/fnbeh.2018.00163 30116182 PMC6082963

[B4] Benson DL, Isackson PJ, Gall CM, Jones EG (1992) Contrasting patterns in the localization of glutamic-acid decarboxylase and Ca2+/calmodulin protein-kinase gene-expression in the rat central-nervous-system. Neuroscience 46:825–849. 10.1016/0306-4522(92)90188-8 1311814

[B5] Beyer F, Münte TF, Göttlich M, Krämer UM (2015) Orbitofrontal cortex reactivity to angry facial expression in a social interaction correlates with aggressive behavior. Cereb Cortex 25:3057–3063. 10.1093/cercor/bhu101 24842782

[B6] Blair RJ (2004) The roles of orbital frontal cortex in the modulation of antisocial behavior. Brain Cogn 55:198–208. 10.1016/S0278-2626(03)00276-8 15134853

[B7] Blanchard RJ, Blanchard DC (1977) Aggressive behavior in the rat. Behav Biol 21:197–224. 10.1016/s0091-6773(77)90308-x 562152

[B8] Buades-Rotger M, Beyer F, Krämer UM (2017) Avoidant responses to interpersonal provocation are associated with increased amygdala and decreased mentalizing network activity. eNeuro 4 10.1523/ENEURO.0337-16.2017 PMC5485378 28660251

[B9] Cádiz-Moretti B, Otero-García M, Martínez-García F, Lanuza E (2016) Afferent projections to the different medial amygdala subdivisions: a retrograde tracing study in the mouse. Brain Struct Funct 221:1033–1065. 10.1007/s00429-014-0954-y 25503449

[B10] Challis C, Boulden J, Veerakumar A, Espallergues J, Vassoler FM, Pierce RC, Beck SG, Berton O (2013) Raphe GABAergic neurons mediate the acquisition of avoidance after social defeat. J Neurosci 33:13978–13988, 13988a. 10.1523/JNEUROSCI.2383-13.2013 23986235 PMC3756748

[B11] Chen C, Rainnie DG, Greene RW, Tonegawa S (1994) Abnormal fear response and aggressive behavior in mutant mice deficient for alpha-calcium-calmodulin kinase II. Science 266:291–294. 10.1126/science.7939668 7939668

[B12] Clarke HF, Walker SC, Dalley JW, Robbins TW, Roberts AC (2007) Cognitive inflexibility after prefrontal serotonin depletion is behaviorally and neurochemically specific. Cereb Cortex 17:18–27. 10.1093/cercor/bhj120 16481566

[B13] Coccaro EF, McCloskey MS, Fitzgerald DA, Phan KL (2007) Amygdala and orbitofrontal reactivity to social threat in individuals with impulsive aggression. Biol Psychiatry 62:168–178. 10.1016/j.biopsych.2006.08.024 17210136

[B14] Commons KG (2009) Locally collateralizing glutamate neurons in the dorsal raphe nucleus responsive to substance P contain vesicular glutamate transporter 3 (VGLUT3). J Chem Neuroanat 38:273–281. 10.1016/j.jchemneu.2009.05.005 19467322 PMC2767471

[B15] da Veiga CP, Miczek KA, Lucion AB, de Almeida RM (2011) Social instigation and aggression in postpartum female rats: role of 5-Ht1A and 5-Ht1B receptors in the dorsal raphe nucleus and prefrontal cortex. Psychopharmacology (Berl) 213:475–487. 10.1007/s00213-010-2083-5 21107539 PMC3747518

[B16] De Almeida RM, Rosa MM, Santos DM, Saft DM, Benini Q, Miczek KA (2006) 5-HT(1B) receptors, ventral orbitofrontal cortex, and aggressive behavior in mice. Psychopharmacology (Berl) 185:441–450. 10.1007/s00213-006-0333-3 16550387

[B17] Golden SA, Heshmati M, Flanigan M, Christoffel DJ, Guise K, Pfau ML, Aleyasin H, Menard C, Zhang H, Hodes GE, Bregman D, Khibnik L, Tai J, Rebusi N, Krawitz B, Chaudhury D, Walsh JJ, Han MH, Shapiro ML, Russo SJ (2016) Basal forebrain projections to the lateral habenula modulate aggression reward. Nature 534:688–692. 10.1038/nature18601 27357796 PMC4930107

[B18] Grafman J, Schwab K, Warden D, Pridgen A, Brown HR, Salazar AM (1996) Frontal lobe injuries, violence, and aggression: a report of the Vietnam Head Injury Study. Neurology 46:1231–1238. 10.1212/wnl.46.5.1231 8628458

[B19] Gunaydin LA, Yizhar O, Berndt A, Sohal VS, Deisseroth K, Hegemann P (2010) Ultrafast optogenetic control. Nat Neurosci 13:387–392. 10.1038/nn.2495 20081849

[B20] Haller J (1995) Biochemical background for an analysis of cost-benefit interrelations in aggression. Neurosci Biobehav Rev 19:599–604. 10.1016/0149-7634(95)00053-4 8684718

[B21] Haller J (2018) The role of central and medial amygdala in normal and abnormal aggression: a review of classical approaches. Neurosci Biobehav Rev 85:34–43. 10.1016/j.neubiorev.2017.09.017 28918358

[B22] Herpertz SC, Nagy K, Ueltzhöffer K, Schmitt R, Mancke F, Schmahl C, Bertsch K (2017) Brain mechanisms underlying reactive aggression in borderline personality disorder-sex matters. Biol Psychiatry 82:257–266. 10.1016/j.biopsych.2017.02.1175 28388995

[B23] Holschbach MA, Vitale EM, Lonstein JS (2018) Serotonin-specific lesions of the dorsal raphe disrupt maternal aggression and caregiving in postpartum rats. Behav Brain Res 348:53–64. 10.1016/j.bbr.2018.04.008 29653128 PMC5993625

[B24] Hong W, Kim DW, Anderson DJ (2014) Antagonistic control of social versus repetitive self-grooming behaviors by separable amygdala neuronal subsets. Cell 158:1348–1361. 10.1016/j.cell.2014.07.049 25215491 PMC4167378

[B25] Huang KW, Ochandarena NE, Philson AC, Hyun M, Birnbaum JE, Cicconet M, Sabatini BL (2019) Molecular and anatomical organization of the dorsal raphe nucleus. Elife 8 10.7554/eLife.46464 PMC6726424 31411560

[B26] Jiao Y, Sun Z, Lee T, Fusco FR, Kimble TD, Meade CA, Cuthbertson S, Reiner A (1999) A simple and sensitive antigen retrieval method for free-floating and slide-mounted tissue sections. J Neurosci Methods 93:149–162. 10.1016/s0165-0270(99)00142-9 10634500

[B27] Jones EG, Huntley GW, Benson DL (1994) Alpha calcium/calmodulin-dependent protein kinase II selectively expressed in a subpopulation of excitatory neurons in monkey sensory-motor cortex: comparison with GAD-67 expression. J Neurosci 14:611–629. 10.1523/JNEUROSCI.14-02-00611.1994 8301355 PMC6576801

[B28] Kollack-Walker S, Newman SW (1995) Mating and agonistic behavior produce different patterns of Fos immunolabeling in the male Syrian hamster brain. Neuroscience 66:721–736. 10.1016/0306-4522(94)00563-k 7644033

[B29] Koolhaas JM, Coppens CM, de Boer SF, Buwalda B, Meerlo P, Timmermans PJ (2013) The resident-intruder paradigm: a standardized test for aggression, violence and social stress. J Vis Exp. Advance online publication. Retrieved July 4, 2013. doi: 10.3791/4367. 10.3791/4367 PMC3731199 23852258

[B30] Kuniishi H, Ichisaka S, Matsuda S, Futora E, Harada R, Hata Y (2016) Chronic inactivation of the orbitofrontal cortex increases anxiety-like behavior and impulsive aggression, but decreases depression-like behavior in rats. Front Behav Neurosci 10:250. 10.3389/fnbeh.2016.00250 28167902 PMC5253363

[B31] Lin D, Boyle MP, Dollar P, Lee H, Lein ES, Perona P, Anderson DJ (2011) Functional identification of an aggression locus in the mouse hypothalamus. Nature 470:221–226. 10.1038/nature09736 21307935 PMC3075820

[B32] Liu XB, Jones EG (1996) Localization of alpha type II calcium calmodulin-dependent protein kinase at glutamatergic but not gamma-aminobutyric acid (GABAergic) synapses in thalamus and cerebral cortex. Proc Natl Acad Sci USA 93:7332–7336. 10.1073/pnas.93.14.7332 8692993 PMC38984

[B33] Liu Z, Zhou J, Li Y, Hu F, Lu Y, Ma M, Feng Q, Zhang JE, Wang D, Zeng J, Bao J, Kim JY, Chen ZF, El Mestikawy S, Luo M (2014) Dorsal raphe neurons signal reward through 5-HT and glutamate. Neuron 81:1360–1374. 10.1016/j.neuron.2014.02.010 24656254 PMC4411946

[B34] Malick JB (1979) The pharmacology of isolation-induced aggressive behavior in mice. Curr Dev Psychopharmacol 5:1–27. 35310

[B35] Márquez C, Poirier GL, Cordero MI, Larsen MH, Groner A, Marquis J, Magistretti PJ, Trono D, Sandi C (2013) Peripuberty stress leads to abnormal aggression, altered amygdala and orbitofrontal reactivity and increased prefrontal MAOA gene expression. Transl Psychiatry 3:e216. 10.1038/tp.2012.144 23321813 PMC3566724

[B36] Matthews GA, Nieh EH, Vander Weele CM, Halbert SA, Pradhan RV, Yosafat AS, Glober GF, Izadmehr EM, Thomas RE, Lacy GD, Wildes CP, Ungless MA, Tye KM (2016) Dorsal raphe dopamine neurons represent the experience of social isolation. Cell 164:617–631. 10.1016/j.cell.2015.12.040 26871628 PMC4752823

[B37] Mattis J, Tye KM, Ferenczi EA, Ramakrishnan C, O'Shea DJ, Prakash R, Gunaydin LA, Hyun M, Fenno LE, Gradinaru V, Yizhar O, Deisseroth K (2011) Principles for applying optogenetic tools derived from direct comparative analysis of microbial opsins. Nat Methods 9:159–172. 10.1038/nmeth.1808 22179551 PMC4165888

[B38] Maynard Smith J, Price G (1973) The logic of animal conflict. Nature 246:15–18. 10.1038/246015a0

[B39] Miczek KA, de Boer SF, Haller J (2013) Excessive aggression as model of violence: a critical evaluation of current preclinical methods. Psychopharmacology (Berl) 226:445–458. 10.1007/s00213-013-3008-x 23430160 PMC3595336

[B40] Miller SM, Marcotulli D, Shen A, Zweifel LS (2019) Divergent medial amygdala projections regulate approach-avoidance conflict behavior. Nat Neurosci 22:565–575. 10.1038/s41593-019-0337-z 30804529 PMC6446555

[B41] Mpakopoulou M, Gatos H, Brotis A, Paterakis KN, Fountas KN (2008) Stereotactic amygdalotomy in the management of severe aggressive behavioral disorders. Neurosurg Focus 25:E6. 10.3171/FOC/2008/25/7/E6 18590383

[B42] Muroi Y, Ishii T (2019) Glutamatergic signals in the dorsal raphe nucleus regulate maternal aggression and care in an opposing manner in mice. Neuroscience 400:33–47. 10.1016/j.neuroscience.2018.12.034 30605702

[B43] Murphy MJM, Deutch AY (2018) Organization of afferents to the orbitofrontal cortex in the rat. J Comp Neurol 526:1498–1526. 10.1002/cne.24424 29524205 PMC5899655

[B44] Nelson RJ (2006) Biology of aggression. Oxford: Oxford University Press.

[B45] Nelson RJ, Trainor BC (2007) Neural mechanisms of aggression. Nat Rev Neurosci 8:536–546. 10.1038/nrn2174 17585306

[B46] New AS, Buchsbaum MS, Hazlett EA, Goodman M, Koenigsberg HW, Lo J, Iskander L, Newmark R, Brand J, O'Flynn K, Siever LJ (2004) Fluoxetine increases relative metabolic rate in prefrontal cortex in impulsive aggression. Psychopharmacology (Berl) 176:451–458. 10.1007/s00213-004-1913-8 15160265

[B47] Nordman JC, Ma X, Gu Q, Potegal M, Li H, Kravitz AV, Li Z (2020a) Potentiation of divergent medial amygdala pathways drives experience-dependent aggression escalation. J Neurosci 40:4858–4880. 10.1523/JNEUROSCI.0370-20.2020 32424020 PMC7326350

[B48] Nordman J, Ma X, Li Z (2020b) Traumatic stress induces prolonged aggression increase through synaptic potentiation in the medial amygdala circuits. eNeuro 7 10.1523/ENEURO.0147-20.2020 PMC7385664 32651265

[B49] Olivier B (2004) Serotonin and aggression. Ann NY Acad Sci 1036:382–392. 10.1196/annals.1330.022 15817750

[B50] Padilla SL, Qiu J, Soden ME, Sanz E, Nestor CC, Barker FD, Quintana A, Zweifel LS, Rønnekleiv OK, Kelly MJ, Palmiter RD (2016) Agouti-related peptide neural circuits mediate adaptive behaviors in the starved state. Nat Neurosci 19:734–741. 10.1038/nn.4274 27019015 PMC4846501

[B51] Puciłowski O, Płaźnik A, Kostowski W (1985) Aggressive behavior inhibition by serotonin and quipazine injected into the amygdala in the rat. Behav Neural Biol 43:58–68. 10.1016/s0163-1047(85)91496-7 4039562

[B52] Qi J, Zhang S, Wang HL, Wang H, de Jesus Aceves Buendia J, Hoffman AF, Lupica CR, Seal RP, Morales M (2014) A glutamatergic reward input from the dorsal raphe to ventral tegmental area dopamine neurons. Nat Commun 5:5390. 10.1038/ncomms6390 25388237 PMC4231541

[B53] Ren J, Friedmann D, Xiong J, Liu CD, Ferguson BR, Weerakkody T, DeLoach KE, Ran C, Pun A, Sun Y, Weissbourd B, Neve RL, Huguenard J, Horowitz MA, Luo L (2018) Anatomically defined and functionally distinct dorsal raphe serotonin sub-systems. Cell 175:472–487.e20. 10.1016/j.cell.2018.07.043 30146164 PMC6173627

[B54] Rodgers RJ (1977) The medial amygdala: serotonergic inhibition of shock‐lnduced aggression and pain sensitivity in rats. Aggr Behav 3:277–288. 10.1002/1098-2337(1977)3:3<277::AID-AB2480030309>3.0.CO;2-5

[B55] Rosell DR, Siever LJ (2015) The neurobiology of aggression and violence. CNS Spectr 20:254–279. 10.1017/S109285291500019X 25936249

[B56] Rosell DR, Thompson JL, Slifstein M, Xu X, Frankle WG, New AS, Goodman M, Weinstein SR, Laruelle M, Abi-Dargham A, Siever LJ (2010) Increased serotonin 2A receptor availability in the orbitofrontal cortex of physically aggressive personality disordered patients. Biol Psychiatry 67:1154–1162. 10.1016/j.biopsych.2010.03.013 20434136 PMC3091264

[B57] Scheyltjens I, Laramée ME, Van den Haute C, Gijsbers R, Debyser Z, Baekelandt V, Vreysen S, Arckens L (2015) Evaluation of the expression pattern of rAAV2/1, 2/5, 2/7, 2/8, and 2/9 serotypes with different promoters in the mouse visual cortex. J Comp Neurol 523:2019–2042. 10.1002/cne.23819 26012540

[B58] Shalom G, Gur E, Van de Kar LD, Newman ME (2004) Repeated administration of the 5-HT(1B) receptor antagonist SB-224289 blocks the desensitisation of 5-HT(1B) autoreceptors induced by fluoxetine in rat frontal cortex. Naunyn Schmiedebergs Arch Pharmacol 370:84–90. 10.1007/s00210-004-0958-x 15309378

[B59] Siever LJ (2008) Neurobiology of aggression and violence. Am J Psychiatry 165:429–442. 10.1176/appi.ajp.2008.07111774 18346997 PMC4176893

[B60] Takahashi A, Miczek KA (2014) Neurogenetics of aggressive behavior: studies in rodents. Curr Top Behav Neurosci 17:3–44. 10.1007/7854_2013_263 24318936 PMC4092042

[B61] Takahashi A, Shimamoto A, Boyson CO, DeBold JF, Miczek KA (2010) GABA(B) receptor modulation of serotonin neurons in the dorsal raphé nucleus and escalation of aggression in mice. J Neurosci 30:11771–11780. 10.1523/JNEUROSCI.1814-10.2010 20810897 PMC2943331

[B62] Takahashi A, Lee RX, Iwasato T, Itohara S, Arima H, Bettler B, Miczek KA, Koide T (2015) Glutamate input in the dorsal raphe nucleus as a determinant of escalated aggression in male mice. J Neurosci 35:6452–6463. 10.1523/JNEUROSCI.2450-14.2015 25904796 PMC6605224

[B63] Unger EK, Burke KJ Jr, Yang CF, Bender KJ, Fuller PM, Shah NM (2015) Medial amygdalar aromatase neurons regulate aggression in both sexes. Cell Rep 10:453–462. 10.1016/j.celrep.2014.12.040 25620703 PMC4349580

[B64] Valzelli L (1985) Animal models of behavioral pathology and violent aggression. Methods Find Exp Clin Pharmacol 7:189–193. 4040594

[B65] Walletschek H, Raab A (1982) Spontaneous activity of dorsal raphe neurons during defensive and offensive encounters in the tree-shrew. Physiol Behav 28:697–705. 10.1016/0031-9384(82)90054-3 7200622

[B66] Watakabe A, Ohtsuka M, Kinoshita M, Takaji M, Isa K, Mizukami H, Ozawa K, Isa T, Yamamori T (2015) Comparative analyses of adeno-associated viral vector serotypes 1, 2, 5, 8 and 9 in marmoset, mouse and macaque cerebral cortex. Neurosci Res 93:144–157. 10.1016/j.neures.2014.09.002 25240284

[B67] Wilson MA, Molliver ME (1991) The organization of serotonergic projections to cerebral cortex in primates: regional distribution of axon terminals. Neuroscience 44:537–553. 10.1016/0306-4522(91)90076-z 1754051

[B68] Zhou J, Jia C, Feng Q, Bao J, Luo M (2015) Prospective coding of dorsal raphe reward signals by the orbitofrontal cortex. J Neurosci 35:2717–2730. 10.1523/JNEUROSCI.4017-14.2015 25673861 PMC6605606

